# Spectral guided sparse inverse covariance estimation of metabolic networks in Parkinson’s disease

**DOI:** 10.1016/j.neuroimage.2020.117568

**Published:** 2020-11-25

**Authors:** Phoebe G. Spetsieris, David Eidelberg

**Affiliations:** a Center for Neurosciences, The Feinstein Institutes for Medical Research, Manhasset, NY 11030, USA

**Keywords:** Principal component analysis (PCA), Sparse inverse covariance estimation (SICE), Graphical lasso (GLASSO), Positron emission tomography (PET), Parkinson’s disease (PD), Metabolic functional connectivity

## Abstract

In neurodegenerative disorders, a clearer understanding of the underlying aberrant networks facilitates the search for effective therapeutic targets and potential cures. [^18^F]-fluorodeoxyglucose (FDG) positron emission tomography (PET) imaging data of brain metabolism reflects the distribution of glucose consumption known to be directly related to neural activity. In FDG PET resting-state metabolic data, characteristic disease-related patterns have been identified in group analysis of various neurodegenerative conditions using principal component analysis of multivariate spatial covariance. Notably, among several parkinsonian syndromes, the identified Parkinson’s disease-related pattern (PDRP) has been repeatedly validated as an imaging biomarker of PD in independent groups worldwide. Although the primary nodal associations of this network are known, its connectivity is not fully understood. Here, we describe a novel approach to elucidate functional principal component (PC) network connections by performing graph theoretical sparse network derivation directly within the disease relevant PC partition layer of the whole brain data rather than by searching for associations retrospectively in whole brain sparse representations. Using sparse inverse covariance estimation of each overlapping PC partition layer separately, a single coherent network is detected for each layer in contrast to more spatially modular segmentation in whole brain data analysis. Using this approach, the major nodal hubs of the PD disease network are identified and their characteristic functional pathways are clearly distinguished within the basal ganglia, midbrain and parietal areas. Network associations are further clarified using Laplacian spectral analysis of the adjacency matrices. In addition, the innate discriminative capacity of the eigenvector centrality of the graph derived networks in differentiating PD versus healthy external data provides evidence of their validity.

## 1. Introduction

In brain function studies using positron emission tomography (PET) and [^18^F]-fluorodeoxyglucose (FDG), images are measures of resting-state glucose metabolism that directly reflect neural synaptic activity (Patel et al., 2014; Stoessl, 2017). In contrast, functional MRI (fMRI) images are derived from indirect BOLD brain signals of changes in local blood flow and oxygenation (Ekstrom, 2010). Further, multivariate analyses of FDG PET data (Eidelberg, 2009; Habeck et al., 2008; Sala and Perani, 2019; Trošt et al., 2019) provide a comprehensive approach to decipher the convoluted neural signals of the brain compared to studying localized brain differences using univariate analysis (Friston et al., 2007). Among multivariate approaches, we have applied spectral decomposition of the covariance matrix, i.e., principal component analysis (PCA) (Jolliffe and Cadima, 2016) of spatial metabolic resting-state FDG PET data using the scaled subprofile model (SSM) (Alexander and Moeller, 1994; Moeller and Strother, 1991), in studies of parkinsonian syndromes in both regional (Eidelberg et al., 1995) and in voxel-based data (Eidelberg, 2009; Ma et al., 2007; Spetsieris and Eidelberg, 2011) to derive disease-specific principal component (PC) patterns. In sharp contrast to ICA-based patterns acquired with resting-state fMRI (Calhoun et al., 2009; Vo et al., 2017), these group in-variant patterns are metabolic imaging biomarkers of each disease (Schindlbeck and Eidelberg, 2018) whose expression in individual subjects is evaluated by a scalar score. The test/retest reliability and broad applicability of SSM-PCA patterns and subject scores have enabled numerous comprehensive studies of parkinsonian and other neurodegenerative conditions in group comparisons as well as single subject diagnosis, patient monitoring and therapeutic appraisals (Eidelberg, 2009; Koet al., 2014, 2015; Mattis et al., 2016; Schindlbeck et al., 2020; Schindlbeck and Eidelberg, 2018; Spetsieris et al., 2015; Spetsieris and Eidelberg, 2011; Tang et al., 2010). In particular, using voxel-based SSM-PCA applied to a specific data set of 33 Parkinson’s disease (PD33) patients spanning various stages of the disease and 33 age-matched, healthy subjects (Ma et al., 2007), the derived primary principal component (PC1) has been repeatedly validated as an imaging biomarker of the disease (PDRP) in world-wide prospective studies (Meles et al., 2020; Rus et al., 2020; Schindlbeck and Eidelberg, 2018). Although the spatial configuration of this pattern has been displayed in detail in orthogonal tomographic slices or surface renderings of voxel weights, the underlying regional functional connectivity (Bullmore and Sporns, 2009) of the PC network is not easily apparent in these maps. In view of this recent increased recognition of the PDRP as a signature pattern and imaging biomarker of PD, we examined the underlying structure of this network more closely using newer approaches to provide further insight as to its atypical pathways. Following graph theoretic methodologies introduced by Borgatti and Everett (2006), Bullmore and Sporns (2009), Göttlich et al. (2013), Newman (2010), and Rubinov and Sporns (2010), we performed an in-depth analysis of the PD33 patient data set using regional correlation data (Ko et al., 2018) to study the interconnectivity of highly weighted positive and negative nodes of the PDRP, their sensitivity to perturbation (Correa et al., 2012), and the characteristics of their graphical parameters exhibited over a wide range of graph density values. This analysis revealed abnormal clustering, reduced path length and disproportionate small-world increases (Humphries and Gurney, 2008; Watts and Strogatz, 1998) as well as a strong correlation of the graph eigenvector centrality (EC) (Lohmann et al., 2010; Meyer, 2000; Newman, 2010; Zuo et al., 2012) of the PD33 adjacency matrix to the absolute regional weights of the PDRP principal component. Further, in a preliminary study (Spetsieris et al., 2018) we used the methodology of the graphical lasso (GLASSO) (Friedman et al., 2008) of sparse inverse covariance (SICE) to estimate the precision matrix and identify prominent functional connections induced by the disease without prior knowledge of the PDRP. Although numerous algorithms have been applied to estimate undirected network models, the GLASSO algorithm has proven to be a reliable popular model that has been successfully employed in numerous previous assessments of connectivity in brain-based analysis. In contrast to previous applications of the GLASSO methodology in neurodegenerative disorders (Huang et al., 2010; Ortiz et al., 2015; Sala et al., 2017; Titov et al., 2017), we introduced the concept of utilizing the partition of the data associated with each principal component separately within GLASSO to visualize the specific major connections of the PD33_PC1 component. Sparse connections and regional thresholding was determined by the GLASSO penalization of the full region PC partition data rather than pre-sparsified as in sparse principal component analysis (Zou et al., 2006).

Here, we expand our previous GLASSO/PCA analysis to further examine the PD33_PC1 partition connectivity and compare it to that of the whole brain PD33 disease group and healthy NL33 group GLASSO network configurations as well as to their PC1 and PC2 partitioned components. The first two PCs of the two groups, were investigated because their high contribution to the overall variance implies a significant effect on overall connectivity in the sparse state. PD33-PC1 was the most discriminative PC and also accounted for the greatest variance in the disease data. Although the PDRP was derived from the combined PD33 and NL33 voxel data, the main features of the pattern result from the variance in the disease group which are also expressed in the PD33 regional data. Results are evaluated under a wide range of sparsity values in extended penalized bootstrap sampling. We further applied Laplacian spectral decomposition (Chung, 1997; Fiedler, 1973; Kelner et al., 2009; Von Luxburg, 2007) to study the functional spatial modularity within the whole brain graphical analysis of the PD33 group prominent connections, and in that of the PD33_PC1 partition of the data. Overall, our application of GLASSO methodology in conjunction with spectral decomposition of the original PD sample, as well as the graph adjacency matrix and the graph Laplacian, revealed specific information regarding functional regional associations, the modularity of the regional clustering, and the connectivity of the dominant hubs that characterize the disease.

## 2. Material and methods

### 2.1. Group demographics

Two main groups of subjects were studied, comprised of 33 PD patients (PD33; 22 m/11 f, age 57.2 ± 8.2 years (mean ± SD), duration 9.2 ± 3.6 years, range [5–18] years), and a group of age-matched healthy subjects (NL33; 10 m/23 f, age 55.0 ± 13.4 years) used in the original derivation of the PDRP (Ma et al., 2007). The Unified Parkinson’s Disease Rating Scale (UPDRS)-III motor rating scores (off medication) varied broadly (32.4 ± 16.7, range [2–72]). The PD33 group was also previously studied using both voxel-based and regional techniques (Ko et al., 2018; Spetsieris et al., 2018, 2015). An additional independent group of 13 PD early stage patients (PD13; 10 m/3 f, age 61.0 ± 6.6 years, duration 1.7 ± 0.9 years) and matched healthy controls (NL13; 8 m/5 f, age 60.3 ± 7.4 years) were assessed in prospective discriminant analysis. Ethical permission for the procedures was obtained from the Institutional Review Board of Northwell Health. Written consent was obtained from each subject following detailed explanation of the scanning procedures.

### 2.2. Data acquisition and software

Patients and healthy controls underwent resting-state FDG PET scans using the GE Advance tomograph (Milwaukee, WI) at the Feinstein Institutes. Studies were conducted after an overnight fast; PD patients were scanned 12 h after their last medication dose. Maps of glucose metabolism were spatially normalized to a common Montreal Neurological Institute (MNI) stereotaxic space using SPM (http://www.fil.ion.ucl.ac.uk/spm).

We performed the Scaled Subprofile Model of principal component analysis (SSM-PCA) using our MATLAB (Mathworks, Natick, MA) based in-house Scan Analysis and Visualization (ScAnVP) software (http://www.feinsteinneuroscience.org/) (Spetsieris et al., 2013; Spetsieris and Eidelberg, 2011). Graphical analysis was performed using the Brain Connectivity Toolbox (BCT, brain-connectivity-toolbox.net) (Rubinov and Sporns, 2010) in conjunction with customized graph theoretical ScAnVP based algorithms and a customized implementation of the graphical lasso available online (graphicalLasso.m, by X. Chen, UIUC). Mean data values were obtained for 95 regions-of-interest (ROIs) whose names and abbreviations are listed in Table S1 and defined in the automated anatomical labeling (AAL) atlas and modifications as in Ko et al. (2018).

### 2.3. SSM-PCA analysis

We have employed the SSM-PCA model (Alexander and Moeller, 1994; Spetsieris et al., 2015; Spetsieris and Eidelberg, 2011) as it applies to regional resting-state data. As opposed to dynamic, time-varying data temporal analysis, the employed resting-state spatial covariance analysis can identify PC patterns that reflect steady-state brain networks. In its typical application, the model includes equally matched groups of patients and healthy controls to enhance variance in spatial parameters. However, in relatively large groups of data the same patterns can be found in the analysis of the single group that incorporates most of the variance (as we have demonstrated in our previous voxel-based analysis of the DMN (Spetsieris et al., 2015)). Here we used regional values to form the data matrix **D** for each group separately. Specifically, using SSM-PCA spatial covariance analysis, the subject by region mean data matrix for each group (**D**) is initially log transformed and then centered with respect to the subject and group mean values. This ensures that multiplicative scalar factors are removed from the data prior to analysis. PCA is performed on the covariance data of each group’s data matrix **D**, to derive a complete set of orthogonal, spatially overlapping principal components PC_k_ and corresponding subject scores Score_jk_ (Fig. S1). The major PC eigenvectors that result from the PCA analysis are assumed to represent separate underlying network partitions of regional brain function. The data vector from any subject *j* (**D**_**j**_) can be described as a sum of the unit normalized PCs weighted by that subject’s scores (Eq. (1)):

(1) $$D_{j}={\text{Σ}}_{\text{k}}{\text{Score}}_{\text{jk}}PC_{k}$$

Thus, only a portion of the subject *j* data **(D**_**jk**_) equal to the principal component **PC**_**k**_ modified by the corresponding subject score (Score_jk_) can be attributed to a specific principal component **PC**_**k**_ (Eq. (2)):

(2) $$D_{j}={\text{Σ}}_{\text{k}}D_{jk},\text{ where }D_{jk}={\text{ Score }}_{\text{jk}}PC_{k}$$

The composite contributions (**D**_**jk**_) over all subjects *j* that are attributed to the specific k^th^ PC are considered here as the **PC**_**k**_ subnetwork data partition **D**_**PCk**_, (Fig. S1). By identifying the PC components that are associated with disease processes versus normal or other sources of variation we can focus our analysis on the more disease relevant partitions of the data. As these can usually be found within the first few PCs representing the major sources of variance, we are generally able to confine our analysis to the first two or three PCs of the disease group. To validate the discriminative capacity of a particular PC or external pattern vector, we can use Eq. (3), resulting from the orthogonality of the PCs, to evaluate prospective subject scores (Eq. (3)):

(3) $${\text{Score}}_{\text{jk}}=D_{\text{j}}^{\text{T}}\cdot PC_{\text{k}}$$

Essentially, this equation indicates that a subject’s score expression for a particular PC pattern is evaluated as the internal product of the subject data vector and the unitized PC vector.

The same equations apply to either voxel or regional data. In voxel-based analysis the PC vectors are visualized by full brain images of the voxel weights that illustrate the unique pattern distribution of positive and negative values for each PC. A similar representation can be obtained for regional PC vectors by mapping the regional weight value of each region to each of the voxels in the space of the corresponding reference atlas region. The resulting individual PC pattern map representations (regional vector maps) are visually comparable to voxel-based pattern representations. Prospective subjects can be tested by evaluating their expression scores as inner products of their regional data vector and the pre-derived PC pattern vector (Eq. (3)), referred to as topographic profile score ratings (TPR) (Spetsieris et al., 2013; Spetsieris and Eidelberg, 2011). Qualitatively comparable scores were obtained (where indicated) by using the image voxel data vectors and PC pattern regional vector maps (vector map TPR). We can test the probability that a particular PC can be attributed to disease-related covariance sources using Student’s *t*-tests to compare patient versus control expression scores for each group. It should be noted that in SSM-PCA, voxel or regional patient scan data are usually combined with healthy control data to enhance variance, thus providing more robust and detailed patterns. However, graph theoretical analysis is regional because of the computational demands of voxel-based analysis and necessarily evaluates single group, rather than combined group data. Thus, to relate results from GLASSO analysis of each group’s regional data set, we performed regional SSM-PCA analysis using the same data set. We used Pearson’s correlation coefficient to evaluate the relationships between PC pattern vectors and graphical centrality regional vectors and between voxel pattern vectors and regional pattern vector maps.

### 2.4. GLASSO whole brain analysis

The GLASSO algorithm allows us to obtain a sparse representation of the inverse covariance matrix (precision matrix), which reflects the salient functional connections of the brain topology dependent on the level of sparsity (Friedman et al., 2008). Its elements represent partial correlations, i.e., a non-zero element *ij* implies that node *i* is “directly” connected to node *j* (i.e., functionally correlated) in the absence of indirect influences from other regions. Zero elements imply that region *i* is conditionally independent of region *j*. In addition to enabling the determination of the most robust functional connections, the GLASSO algorithm provides an efficient solution to the problem of inverse covariance matrix determination, which is often ill-posed given that the number of subject samples (observations) is far less than the number of variable parameters (regional nodes).

The inverse covariance matrix Θ is estimated by maximizing the penalized Gaussian log-likelihood of the data using the empirical covariance matrix S (Eq. (4)). The level of sparsity is modulated by the regularization parameter *ρ*:

(4) $$\log\det\text{Θ}-\text{tr}(\text{SΘ})-\rho ∥\text{Θ}{∥}_{1}$$

where S is the empirical covariance matrix, Θ=Σ^−1^ is the inverse covariance matrix, tr denotes the matrix trace and ∥Θ∥_1_ is the L_1_ norm (the sum of the absolute values of the elements of Σ^−1^).

The magnitudes of the sparse inverse covariance elements are not considered reliable measures but are used to determine the 0/1 binarized adjacency matrix. The non-zero elements of the adjacency matrix determine the edges of regional nodes in the corresponding graphical representation. Using the adjacency matrix, we can visualize whole brain or subnetwork connectivity depending on whether data from the whole brain or the PC partition (PC subnet layer) are entered into the algorithm. Whole brain analysis is performed at or near the maximum sparsity (defined below) that ensured a fully connected graph involving all or most nodes (at least 90 nodes or 95%) in the whole data derivation samples. Different graph theoretical parameters and centralities (Borgatti and Everett, 2006; Rubinov and Sporns, 2010) are derived from the adjacency matrix as described below.

#### 2.4.1. GLASSO/PCA subnet layer analysis

For subnet analysis, the empirical covariance matrix S in Eq. (4) is the regional covariance of the partition data (**D**jk) (specified by Eq. (2)) determined separately over all subjects *j* for each **PC**_**k**_ of the NL33 and PD33 groups. The GLASSO outcome is not affected by the sign of the PC in accordance with Eqs. (2) and (3). Subnets derived within the specified whole brain maximal sparsity range do not necessarily involve all of the nodes of the full brain as they reflect the regions that are actively interconnected within a specific network. Thus, the connections of the low weighted regions of the PC are suppressed in the penalized graphical analysis, resulting in a smaller number of regions within the connected subnet. The algorithm determines the connected subnet in a data-driven way based on the penalty parameter. As the penalty parameter increases, additional edges are suppressed and the network may also involve fewer regions. Thus, although the initial input data applied to the GLASSO algorithm involves all 95 regions, the algorithm may reduce the effective network to a smaller number of densely connected regions. Thus, the resulting subnet sparsity (nSP) evaluated for the determined connected regions is as a rule lower than the whole brain sparsity (wSP) evaluated for the initial 95 regions. In bootstrap analysis, a range of wSP values is considered for each sample but graphical parameters are evaluated within the resulting nSP range of the connected subnets.

### 2.5. Graph theoretical analysis

Graph theoretical parameters for the whole brain and for subnet sparse fully connected adjacency matrices were evaluated using the BCT toolbox (http://www.brain-connectivity-toolbox.net/) as defined by Rubinov and Sporns (2010) and others. Because of the variable number of fully connected nodes in each network resulting from the disconnection of weaker connections at high sparsity, normalized graphical values in the range [0 1] are evaluated to facilitate comparison between whole brain and subnet configurations. We examined degree and eigenvector centralities as well as other parameters for different sparsity levels. For whole brain data, values were obtained at the maximum sparsity that insured a fully connected graph involving all nodes in the data samples, and at sparsity levels of 85% to 95% in the whole brain and higher sparsity in PC subnet bootstrap data. Sparsity refers to the percent reduction of connections in the network. For an undirected graph, the percent sparsity is given by the following expression:

(5) $$\text{ Sparsity }=100\left(1-\frac{2E}{N(N-1)}\right)$$

where *E* is the number of edges and *N* is the number of nodes.

Within the whole brain configuration, *N* = 95 and we refer to Sparsity as wSP, while within the connected space the actual number of connected nodes may be less (*N* ≤ 95) and we refer to Sparsity as nSP.

In the text, sparsity refers to wSP values for both full brain and subnets unless otherwise stated. In this way, the measure can be used to determine the range of increasing values for the evaluation of full brain networks and subnets with an equivalent numbers of edges. However, graphical parameters for degree centrality (DC), eigenvector centrality (EC), betweenness (BT) and clustering coefficient (CLC) are always determined in the connected space of the adjacency matrix.

The EC vector is defined as the dominant eigenvector of a fully connected adjacency matrix (Perron vector) and has strictly positive elements (Meyer, 2000). High EC nodes represent important graphical hubs (Lohmann et al., 2010; Zuo et al., 2012). A node’s EC weight is related to the number of its important high-weighted node connections, thus reflecting its influence within the network. The EC vector values typically correlate with the absolute SSM PC vector region weights to a varying degree in the order of PC importance (i.e., descending eigenvalue). These values are therefore usually better correlated with the primary PC in whole brain sparse data (Ko et al., 2018), but relate more to a specific primary or secondary PC when the network is derived from that PC’s subnet data (Spetsieris et al., 2018). Here, we also considered signed EC vectors, where the + /− polarity of each node is made to match that of the PC vector from which the subnet is derived or that which correlates more in terms of absolute value. In signing the EC to correspond with the PC sign, we add relevant similarity information in the calculation of the Pearson’s correlation. Matched EC/PC signing was performed to test for topological similarity and not to alter the graphical significance of the EC.

### 2.6. Graph Laplacian

We investigated graphical partitioning of the sparse adjacency matrices in the whole brain and subnet space based on the spectral decomposition of the Laplacian matrix (Chung, 1997; Fiedler, 1973; Kelner et al., 2009; Von Luxburg, 2007). The graph Laplacian matrix is defined as the diagonal degree matrix minus the adjacency matrix; its eigenvalues and eigenvectors provide useful information regarding the number of connected components, spectral clustering, and the graphical parameters of the component modules. Its second smallest eigenvalue corresponding to the second eigenvector (Fiedler vector) reflects the connectivity of the graph and spatial associations of nodes and edges. The nodal weights of the Fiedler vector, when ordered by magnitude, sequence nodes according to their modular associations. The Fiedler ordered nodal index vector was used to sort and redisplay the adjacency matrix (Paulino et al., 1994) thereby shedding light on the modular partitioning of the whole brain images, and the topological structure of the PC subnetworks.

### 2.7. Bootstrap analysis

We performed bootstrap processing with replacement (Efron and Tibshirani, 1998) in 200 to 500 resampled data sets for the NL33 healthy and PD33 patient groups in whole brain as well as in the PD33_PC1 and PD33_PC2 subnet analysis, over a wide range of penalty values for each sample. Hundreds of iterations were performed at each value of the penalty parameter to test for convergence under the GLASSO algorithm (Friedman et al., 2008). Close to 8000 converging cases for different penalty values (about 2000 cases for each of the four data sets) resulted in connected adjacency matrices over the specified sparsity range; these were collectively assessed to test replicability and determine the most robust connections in the PD and healthy control samples. As noted above, for whole brain analysis, a level of 85% to 95% wSP was considered within the full 95 ROI network and higher wSP was tested for subnet analysis in view of substantially higher density (lower nSP) observed within the connected subnet space. For whole brain data, adjacency matrices were within the specified range in 1730 cases for 200 NL33 subject samples and 2319 cases for 200 PD33 subject samples. For partition data, there were 2015 cases for PD33_PC1 in 500 PD33 subject samples and 1932 cases for PD33_PC2 in 400 PD33 subject samples. EC and DC vectors of the connected network within the corresponding output adjacency matrices were evaluated for similarity with the PC derivation and auxiliary network PC vectors by determining the corresponding correlation coefficients. Absolute value correlation and signed EC/PC correlation was plotted for each case to determine continuous ranges of whole brain and subnet sparsity values, over which correlations were stable. The results were used to partition the space for high sparsity and relatively high lower sparsity network visualization. Two and three dimensional representations of prominent connections that occurred in 95% of cases for each group were derived for both the high and lower sparsity bootstrap ranges.

### 2.8. Methodological overview

An initial regional SSM-PCA analysis of our PD disease data group PD33 is performed to determine the PC component whose expression scores best discriminates patient groups from healthy controls. Healthy group NL33 PC components are also derived for comparison. Whole brain GLASSO/SICE analysis is performed in parallel to the PCA analysis to determine and compare the most robust functional connections in the disease and healthy groups based on the sparsest adjacency matrices that ensure full regional connectivity. These parallel but separate operations on the same data are illustrated in the left and right branches of the supplemental flowchart Fig. S1A. In the derivation of the PCA partition subnetworks, summarized in flowchart Fig. S1B, the output PC partition data derived in the PCA operation is input into independent GLASSO procedures to derive network connections for each of the PC subnet partitions separately for a range of penalty parameters. This GLASSO/SICE analysis is performed on specific PC partition data to obtain sparse representations of the sub-network connectivity in specific network layers and determine how they relate to overall connectivity. The EC vector of the adjacency matrix is derived in each case to determine the relative influence (weight) of each regional node in the corresponding sparse configuration. Nodes for which DC or EC are several standard deviation above the mean may be considered as global hubs and are identified by an analogous number of asterisks (*) in Table S2 indicating the deviation of each graphical metric. High correlation of the EC to the absolute PC vector weights indicates that the graph theoretical representation reflects the underlying network associations of the PC nodes. Thus, influential nodes in the GLASSO/SICE network configuration can be assumed to exhibit relatively high positive or negative metabolic activity. The correlation becomes more evident when the EC vector regions are affectedly signed similarly to those of the signed PC vector. The reordering of the adjacency matrix nodes in accordance with the magnitude of the elements of the Laplacian Fiedler vector is performed to reveal the graphical modular structure of both the whole brain and PC partition nodal associations. Bootstrap analysis enables validation of the main findings of the original sample and the delineation of the most robust connections. Within the prominent, sparse configuration, the nodal connectivity pathways of major disease hubs of the network can be traced. As added evidence of the validity and disease specificity of these representations, the discriminant potential of EC vector subject data scores were evaluated in independent group comparisons.

### 2.9. Data availability statement

Deidentified data will be made available on reasonable request at our website (http://www.feinsteinneuroscience.org/) from interested investigators for the purpose of replicating results.

## 3. Results

### 3.1. SSM-PCA analysis

**Fig. 1.**
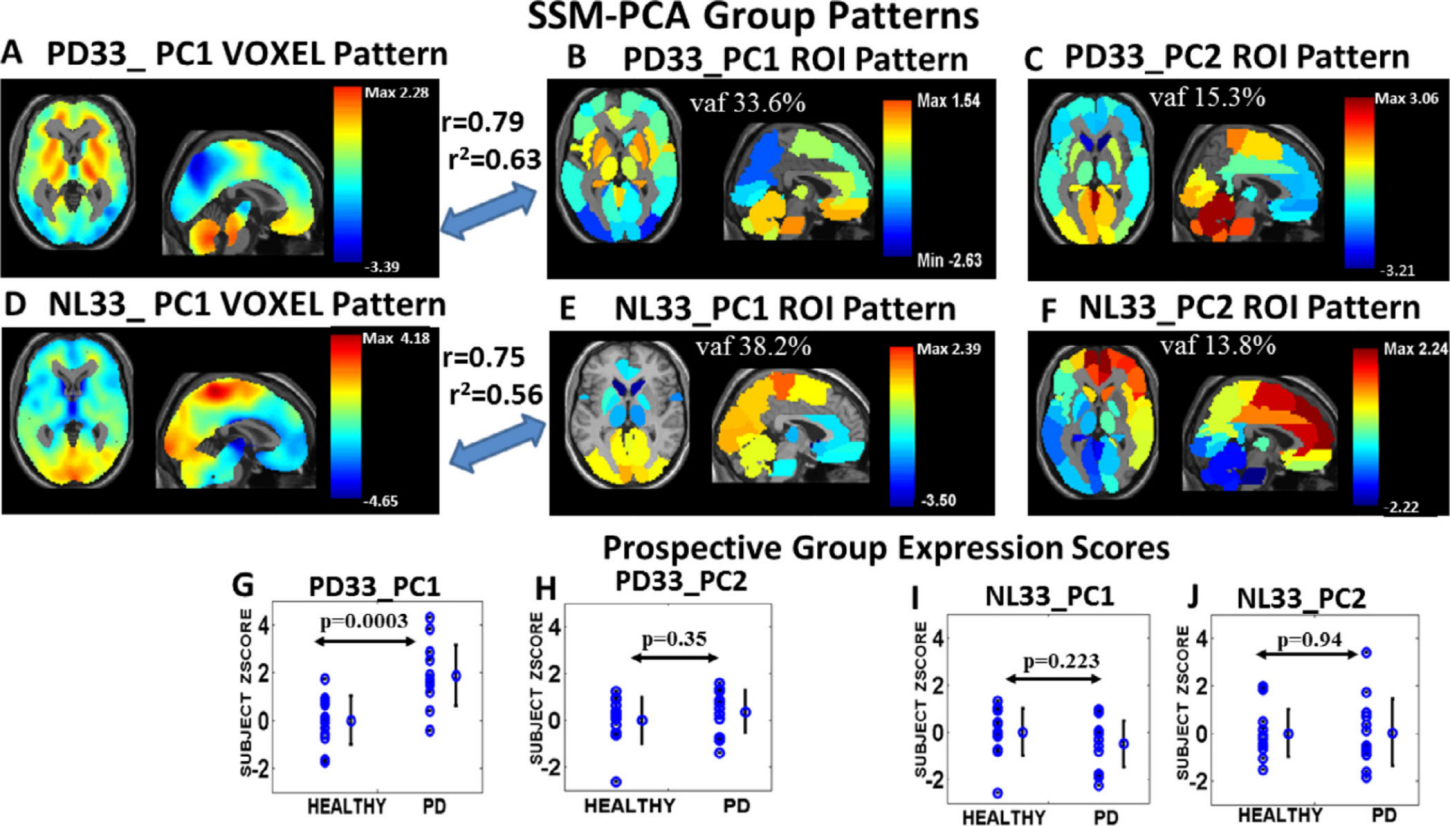
SSM-PCA derivation group PC pattern maps and prospective group scores. Axial and sagittal orthogonal tomographic views of SSM-PCA PC1 and PC2 overlapping patterns derived from the PD33 group data (*top*) and from the NL33 healthy data (*middle*) are shown in the top two rows overlaid on an anatomical MRI background. Voxel data-based patterns (*left* ***A***, ***D***) are shown here for PC1 and mean regional data PC vector maps are shown for PC1(*center*) and PC2 (*righ*t). The correlation between the voxel derived patterns and the corresponding regional pattern maps is shown for PC1 (*r* = 0.79, *p* < 0.001, for PD33_PC1 and *r* = 0.75, *p* < 0.001, for NL33_PC1). The color maps indicate relatively higher metabolic weight in warm color areas and lower weight in cool color areas within the corresponding PC partition layer of the data. *Bottom row:* Prospective subject scores for each of the regional patterns indicating high discrimination for PD33_PC1 (***G***, *p* < 0.0003) between early PD patient (PD13 group) and healthy subjects (NL13 group).

We examined the first two PC patterns of the SSM-PCA analysis of the NL33 and of the PD33 group FDG PET data that accounted for close to 50% of the variance (vaf) in each group and reflect different major network pathways in PD and healthy data. The vaf values for PD33 for the regional PC1 and PC2 were 33.6% and 15.3%, respectively and 38.2% and 13.8%, respectively for NL33_PC1 and NL33_PC2. Axial and sagittal orthogonal tomographic views are shown here for PC1 and PC2 in both regional data sets overlaid on an MRI background (Fig. 1 B, C, E, F). A strong correlation exists between the regional pattern maps and the corresponding voxel derived patterns for PC1 (*r* = 0.79, *p* < 0.001, for PD33_PC1 (Fig. 1 A, B) and *r* = 0.75, *p* < 0.001, for NL33_PC1 (Fig. 1 D, E); Pearson’s correlation). The color maps indicate relatively higher metabolic weight in warm color areas and lower weight in the cool colors within the corresponding PC partition layer of the data. PC signed weights reflect relative values of metabolic components and not absolute metabolism that is a composite of the overlapping PC contributions as expressed by Eq. (1). For PD33_PC1, high values are indicated for the striatum, pons and vermis and low values in parietal and occipital areas. For PD33_PC2 and NL33_PC1, high values were noted for cerebellum and motor areas and low values for the caudate. NL33_PC1 reflects high activity in the sensorimotor network, including the cerebellum and paracentral areas, which is typical of normal metabolic topography. In fact, there is a significant correlation between the PD33_PC2 and NL33_PC1 pattern vectors (*r* = 0.62, *p* < 0.001), while the PD33_PC1 pattern vector correlated negatively with NL33_PC1 (*r* = −0.58, *p* < 0.001). These findings suggest that the original healthy NL33_PC1 topography is a composite of separate partitions that are either resistant to the disease or are affected negatively by it. The NL33_PC2 secondary pattern was characterized by high values for the caudate, frontal areas, and posterior cingulum and demonstrated topographic similarity (*r* = 0.61, *p* < 0.001) with our previously reported metabolic default mode network (DMN) topography (Spetsieris et al., 2015). The bottom row (Fig. 1G–J) indicates the discriminative capacity of each of these regional patterns in prospectively computed subject scores for the independent group of NL13 and PD13 subjects. Only the PD33_PC1 regional pattern significantly discriminated these early PD patients from the healthy group (Figs. 1G and S2A, *p* < 0.0003, AUC = 0.89), thus identifying the primary PD33 pattern as having the greatest disease associations. Discrimination between the derivation PD33 group and the NL33 healthy group scores was, as expected, also significant (*p* = 6.5 × 10^−7^, AUC = 0.85, Fig. S2A). By comparison (Fig. S2B) for the PD33_PC1 voxel pattern (Fig. 1A), significant group discrimination was present for both the derivation (*p* < 9.6 × 10^−8^, AUC = 0.89) and prospective (*p* < 3 × 10^−5^, AUC = 0.94) sets and even greater group discrimination was obtained for the two sets, respectively, with the voxel PDRP pattern (Fig. S2C) (*p* < 6.6 × 10^−12^, AUC = 0.98 and 4.8 × 10^−6^, AUC = 0.97). The receiver operating area under the curve (AUC) values (Fig. S2) for the regional pattern were favorably comparable with those for the voxel patterns as well as for those published for independent PDRP-like voxel patterns ranging from AUC = 0.76 to AUC = 0.96 in several European datasets (Meles et al., 2020).

### 3.2. SICE analysis

We performed GLASSO/SICE analysis for the whole brain signal of the NL33 and PD33 group data and separately for the corresponding PC1 and PC2 partition data of each group.

#### 3.2.1. Whole brain SICE of original samples

**Fig. 2.**
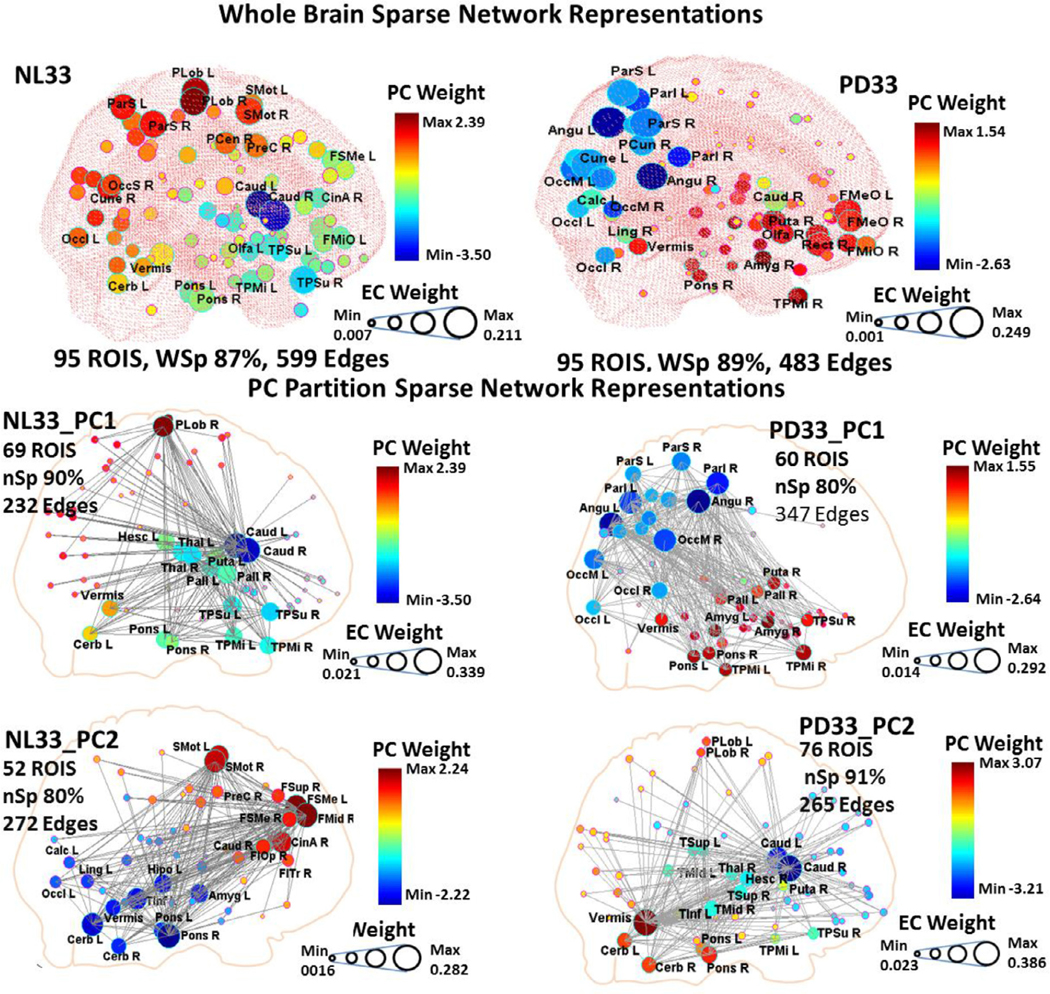
Sparse network three dimensional representations of original sample data. *Top:* Whole brain GLASSO derived nodal configurations for fully connected networks at maximum sparsity are displayed within the three dimensional space of a see-through brain for the NL33 healthy group (*left*) and the PD33 disease group (*right*) original data set. Connection edges are only shown within corresponding supplementary circular displays (Fig. S4, *left*). The diameters of the nodes reflect the EC weights of the corresponding adjacency matrix while the colors correspond to the regional weights of the PC that exhibits the highest absolute value correlation to EC vector weights. *Bottom:* Sparse network configurations are depicted for the partition layers of the first two PCs for the NL33 group (*left*) and PD33 (*right*) derived at specific values of the GLASSO penalty parameter and in circular displays (Fig. S4, *right*). Each of these PC networks exhibited distinct neuronal pathways with central hub nodes strongly linked to a subset of the whole brain regions.

Whole brain GLASSO derived adjacency matrices of salient functional connections for fully connected networks at maximum sparsity of the original samples are displayed for NL33 (Fig. S3, *top*) and PD33 (Fig. S3, *bottom*). Their corresponding nodal configurations within the three dimensional space of a see-through brain are depicted in Fig. 2 for the NL33 healthy group (*top left*) and the PD33 disease group (*top right*). In the 3D displays, the diameters of the nodes reflect the EC weights of the corresponding adjacency matrix, while the colors were selected to correspond to the regional weights of the data’s PC that exhibited the highest absolute value correlation with EC vector weights. Because of the complexity imposed by the high number of edges, we present the connecting edges for the whole brain networks (points of the adjacency matrices) in 2D circular displays (Fig. S4). As visually suggested, the overall graph is thus a composite of highly connected edges dominant in all of the component networks.

For the NL33 configuration (Fig. 2, *top left*) it is clear that there is high EC correlation with NL33_PC1 absolute region weights reflected in dark red and blue regions with high diameter, (rEC = 0.72, rDC = 0.69, *p* < 0.001; Pearson’s correlations) and to a lesser degree with NL33_PC2 absolute regional weights (lighter colors have moderately high diameter), (rEC = 0.36, rDC = 0.38, *p* < 0.001). (See also Fig. S5 for representations of the NL33 network in both NL33_PC1 and NL33_PC2 color schemes.) A better conception of the correspondence between the sparse network and PC representation may be obtained by considering the correlation of the signed PC weights with simulated EC weights that are signed similarly to the PC weights. Signed correlation for these overlapping patterns was higher than for absolute values (rECsigned = 0.90 for NL33_PC1 and rECsigned = 0.83 for NL33_PC2, *p* < 0.001).

**Fig. 3.**
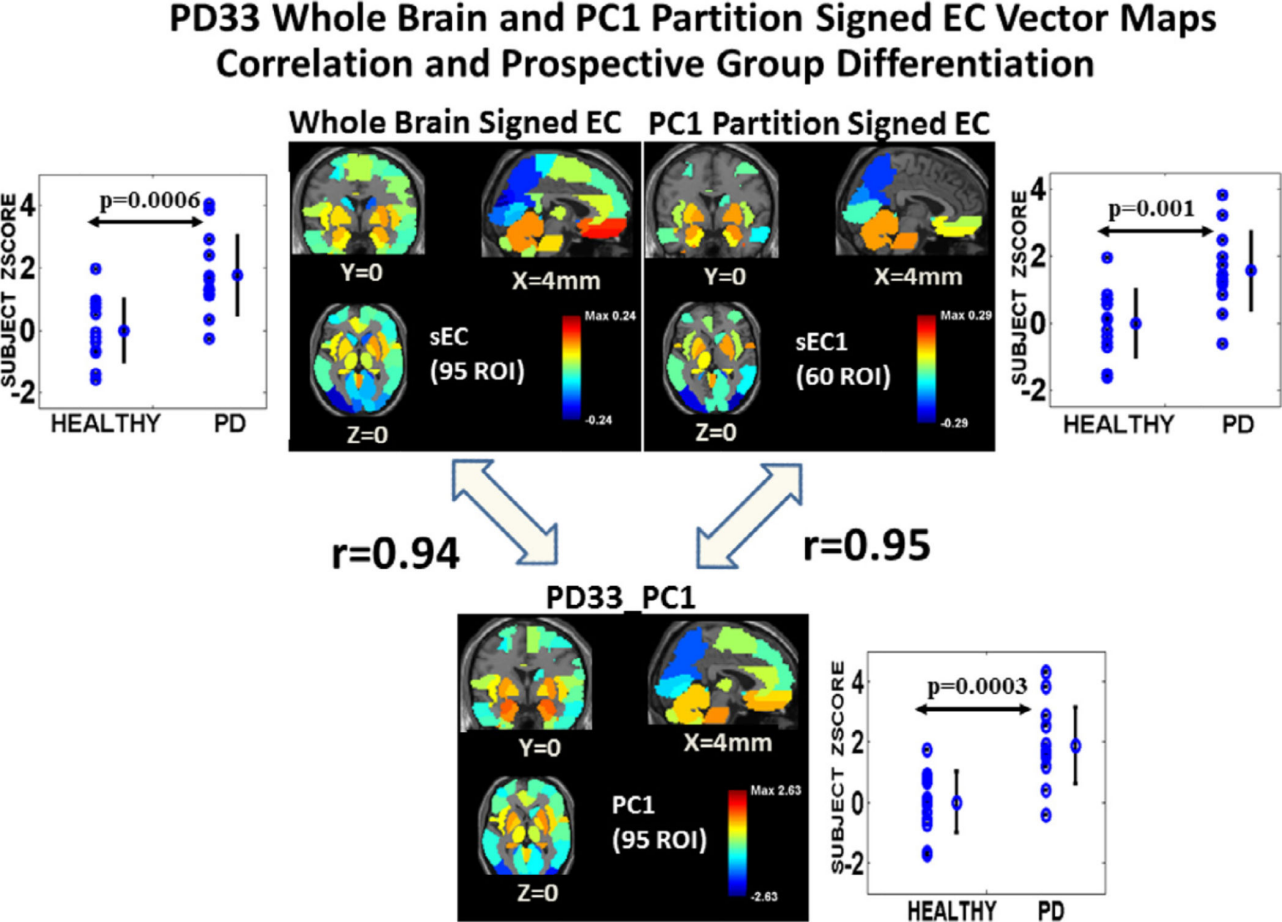
PD33 whole brain and PC1 partition signed EC vector maps of original sample: Correlation to PD33_PC1 and prospective group differentiation. *Top:* The *center left* image represents the signed EC vector nodal weights of the 95 ROI PD33 whole brain adjacency matrix of the original sample mapped to corresponding voxel space regional areas. Scores for this vector pattern (*top left*) significantly discriminated the prospective NL13, PD13 group (*p* = 0.0006). The *center right* image is the vector map of the signed EC vector weights of the PD33_PC1 partition space for 60 ROIs. Prospective discrimination for this vector pattern (*top right*) was lower (*p* = 0.001) but significant. *Bottom:* The *lower* image is a vector map of the PD33_PC1 region weights. Correlation of the whole brain signed EC vector (*top left*) with the PD33_PC1 vector was high (*r* = 0.94, *p* < 0.001; Pearson’s correlation), whereas it was low for the secondary component, PD33_PC2 (*r* < 0.2, *p* > 0.2; see text). *Right:* Correlation of the PD33_PC1 vector with the signed EC vector (*top right*) derived in the PD33_PC1 60 ROI partition space (*r* = 0.95, *p* < 0.001) was slightly higher than for the EC of the whole data signal (*top left*).

For the PD33 whole brain configuration (Fig. 2, *top right*), there is high correlation of EC to PD33_PC1 absolute weights clearly reflected in the correspondence of nodal diameter and color intensity (rEC = 0.84, rDC = 0.76, *p* < 0.001) while the influence of the PD33_PC2 network (Fig. 2, *green areas*) is less evident, (rEC = 0.12, *p* = 0.23, rDC = 0.38, *p* < 0.001). A vector pattern map reflecting signed EC whole brain correlation with PD33_PC1 is depicted in Fig. 3 (*top left* and *bottom center*). Higher correlation is noted for signed PC weights with similarly signed EC weights (rECsigned = 0.94, *p* < 0001 for PD33_PC1 and rECsigned = 0.68, *p* < 0.0001 for PD33_PC2). Subject scores for the whole brain PD33 EC signed vector pattern (Fig. 3, *top left*) significantly discriminated the prospective NL13, PD13 group (*p* = 0.0006).

#### 3.2.2. Subnet SICE of PC partitions of original samples

Similarly, sparse network configurations are depicted for the partition layers of the first two PCs for the NL33 group (Fig. 2, *middle and bottom left*) and PD33 (Fig. 2, *middle and bottom right*). Full network connectivity was not attainable in the sparse state but a connected subnetwork was evident for incremental values of the GLASSO penalty parameter corresponding to varying levels of sparsity. As the degree of sparsity increased, lower weighted nodal connections were eliminated; however, the major hubs and their main connections remained consistent for a wide range of sparsity values in each case. These networks exhibited distinct patterns of nodal associations whose EC vectors were generally highly correlated with the corresponding PC derivation region weights. Characteristically, these networks reflected neuronal pathways linked to one or more central hub nodes. Thus, NL33_PC1 (Fig. 2, *middle left*) links the caudate nuclei and basal ganglia with the pons, vermis and cerebellar hemispheres, temporal poles and the paracentral lobule. NL33_PC2 (Fig. 2, *bottom left*) reflects connectivity between frontal regions, pons, cerebellar hemispheres and vermis, as well as supplementary motor and parietal regions. The disease pattern PD33_PC1 (Fig. 2, *middle right*) links the basal ganglia (largely excluding the caudate) with the pons, vermis, and the limbic system, including temporal poles, as well as the angular gyri, parietal and occipital regions. Correlation with the EC vector within the PD33_PC1, 60 ROI partition space (rEC =0.90, rECsigned = 0.95, rDC = 0.89, *p* < 0.001) was slightly higher than for the EC of the 95 ROI whole data signal (Fig. 3, *top right* and *bottom center*). Prospective discrimination for the PC1 partition signed EC vector pattern (Fig. 3, *top right, p* = 0.001) was lower than for whole brain.

The secondary pattern PD33_PC2 (Fig. 2, *bottom right*) links the caudate with the vermis and the paracentral lobule similar to the healthy NL33_PC1 pattern — excluding the basal ganglia but with the vermis as an interconnecting hub region. For the PD33_PC2 pattern, correlation with the EC vector in its corresponding partition space was also high, although lower than was observed for the primary PD33_PC1 vector (rEC = 0.76, rECsigned = 0.91, rDC = 0.81, *p* < 0.001). For the primary healthy pattern NL33_PC1 correlation of absolute values of NL33_PC1 with EC was low (rEC = 0.58, rECsigned = 0.85, rDC = 0.75, *p* < 0.001) even at high sparsity. For this network, the basal ganglia exhibit low metabolic values but have a high EC index because of their close association with the high EC caudate hub. Because of the negative polarity of the caudate in this network (Fig. 1E), the signed correlation values are more indicative of the association between the PC and the graphical network. By contrast, the NL33_PC2 graphical pattern EC vector was highly correlated with the corresponding PC pattern (rEC = 0.99, rECsigned = 0.97, rDC = 0.92, *p* < 0.001).

### 3.3. Laplacian representation of GLASSO/SICE adjacency

**Fig. 4.**
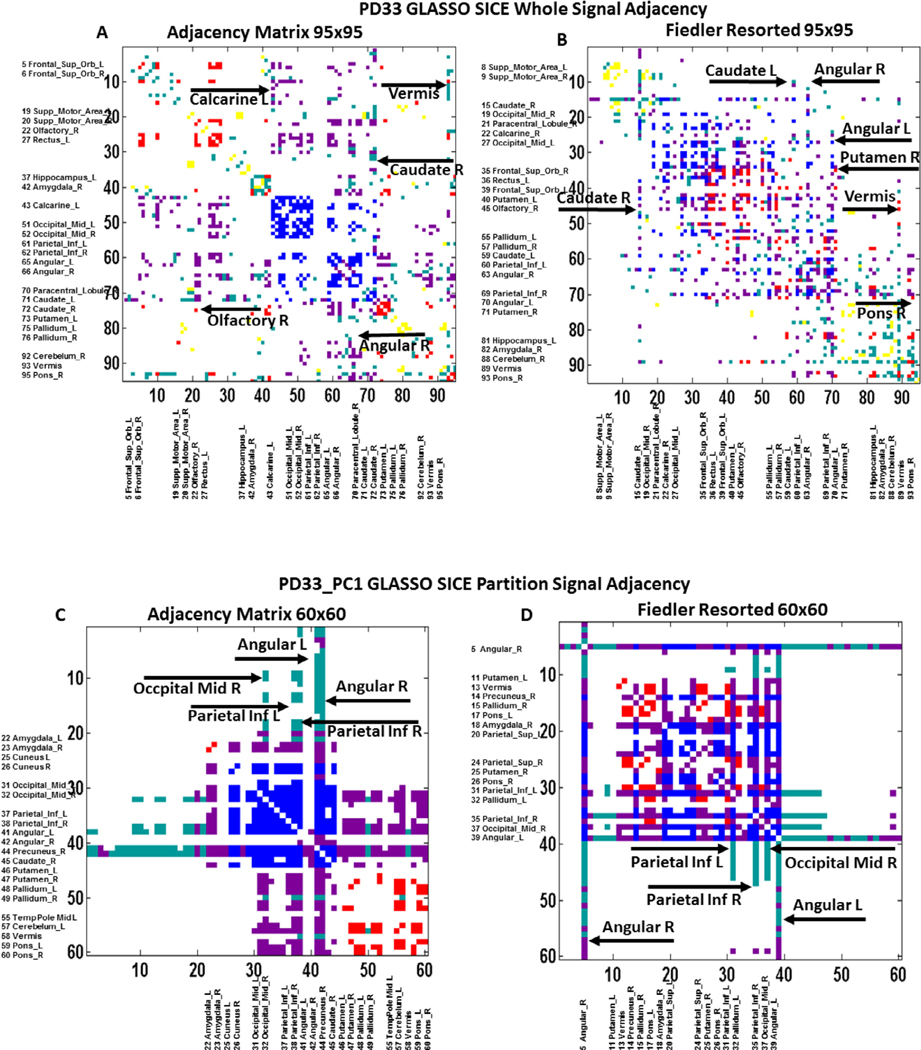
PD33 whole brain and PD33_PC1 partition unsorted and Fiedler resorted adjacency matrices. *Top* : PD33 whole brain adjacency matrix at maximum sparsity for full connectivity for axes ordered in line with the MNI atlas nodal index (*left*) and resorted in accordance with the magnitude of the Fiedler vector nodal weights (*right*). *Bottom*: PD33_PC1 partition data adjacency matrix (*left*: atlas index ordered) and (*right*: Fiedler index resorted). The point elements of these matrices (edges) are colored based on whether they connect high EC (EC ≥ 1) nodes and whether the polarity of the two nodes within the group-related most correlated PC vector was positive or negative. Thus, connections linking high EC positive nodes are shown as red, those between high EC negative nodes are shown in blue; connections linking positive to negative high EC nodes are shown in magenta. Connections linking positive or negative high EC to low EC nodes are depicted in cyan. Connections between low EC nodes are depicted in yellow (none). Vertical and horizontal lines of points indicate the edges of single prominent nodes that act as local or global hubs connecting multiple other nodes.

The graph representations of the GLASSO adjacency matrices of the whole brain analyses are presented in Fig. S3 for the NL33 group (*top*) and PD33 group (*bottom*), and in Fig. 4 for the PD33 whole brain (*top*) and PD33 PC1 partition data (*bottom*). The axes are numbered in line with the nodal atlas index (*left*) or resorted in accordance with their nodal magnitude on the corresponding Fiedler vector (*right*) of the graph Laplacian matrix. Each point represents an edge connecting a node on one axis to a node on the other axis. Thus, each of the regional nodes is represented by a single vertical and horizontal line of points corresponding to its connecting edges to other nodes. Major hubs are noted in Table S2 and on Fig. 4 for the PD33 whole brain and PD33_PC1 adjacency matrices. The resorting of the adjacency matrix based on the nodal magnitude order of the Fiedler vector provides insight as to the modularity of the underlying connective associations. Although PC partitions are not considered a priori for the whole signal GLASSO derivations of the adjacency matrices, the subsequent color mapping of graph points (edges) based on both the magnitude of EC and the weight of the connected nodes of each edge in a specific PC can elucidate the relation of the graph to that PC. Thus, three partially separate modules of edges were detected in the Fiedler resorted adjacency matrix of the PD33 whole brain signal (Fig. S3, *bottom right*) but their association with PD33_PC1 (the PC that is most highly correlated with the whole brain graph EC vector) is not clear in this representation.

Subsequent coloring of the graph points (edges) based on the weights of the PD33_PC1 of the connected nodes (Fig. 4B) as described in the Fig. 4 legend, suggests that the central module spanning reassigned nodes 15 to 71 includes primarily connections of highly weighted PD33_PC1 nodes. This central module includes connections of the basal ganglia and parietal regions that are prominent connections of the PD33_PC1, and is bordered on the left side by the node 15 line (connections of the right caudate) and on the right side by the line of the connections of the left angular gyrus (node 70) and right putamen (node 71). The right side nodes (70 and 71) are also connected with the right module, nodes 70 to 95 that include connections of the pons, cerebellum and vermis regions that are prominent in PD33_PC2, while nodes 1 to 15 involves the right caudate connections to frontal and motor regions that are components of PD33_PC2 and PD33_PC3 (not shown). These outer modules are suppressed (low EC) and, furthermore, are not exclusively independent (not sharply separated). The bordering nodes 15 (right caudate) and 71 (right putamen), represented by intermittent lines, connect the central module with multiple nodes of the left and right module, respectively. In addition, several nodes appearing in the space external to the modules, link individual nodes within the separate modules. These include links of the left caudate and right angular gyrus. Thus, it appears that the whole brain sparse configuration is an integrated representation of the prominent nodal connections associated with all three PCs that are linked at multiple points by hub nodes spanning multiple networks.

On the other hand, the unsorted representation of the adjacency matrix of the PD33_PC1 partition data connected subnet (Fig. 4C) indicates distinct submodules of interconnected negative (blue on blue) nodal edges (within parietal and occipital areas) and red connections (within the basal ganglia, cerebellum, vermis and pons). The red and blue edge modules are strongly linked by distinct modules (magenta) of nodal connections between red and blue nodes. In addition, nodes represented by solid lines serve as hubs linking all nodes including links to low EC nodes (EC < 1std) indicated by cyan. The sorted representation of the PC1 partition adjacency matrix (Fig. 4D) based on the Fiedler vector of the Laplacian matrix, illustrates the existence of a single strongly connected central cluster (upper left rectangular area) including most of the red, blue and magenta connections, bordered by the hub region connections (angular L, R) extending through to the peripheral low EC nodes (cyan points). The inferior parietal nodes (L, R) and occipital mid R nodes also exhibit extended connections including low EC regions. Notably, none of the connections of the PC partition adjacency matrix are between low EC nodes (yellow points).

### 3.4. EC/PC correlations in GLASSO/SICE bootstrap samples

**Fig. 5.**
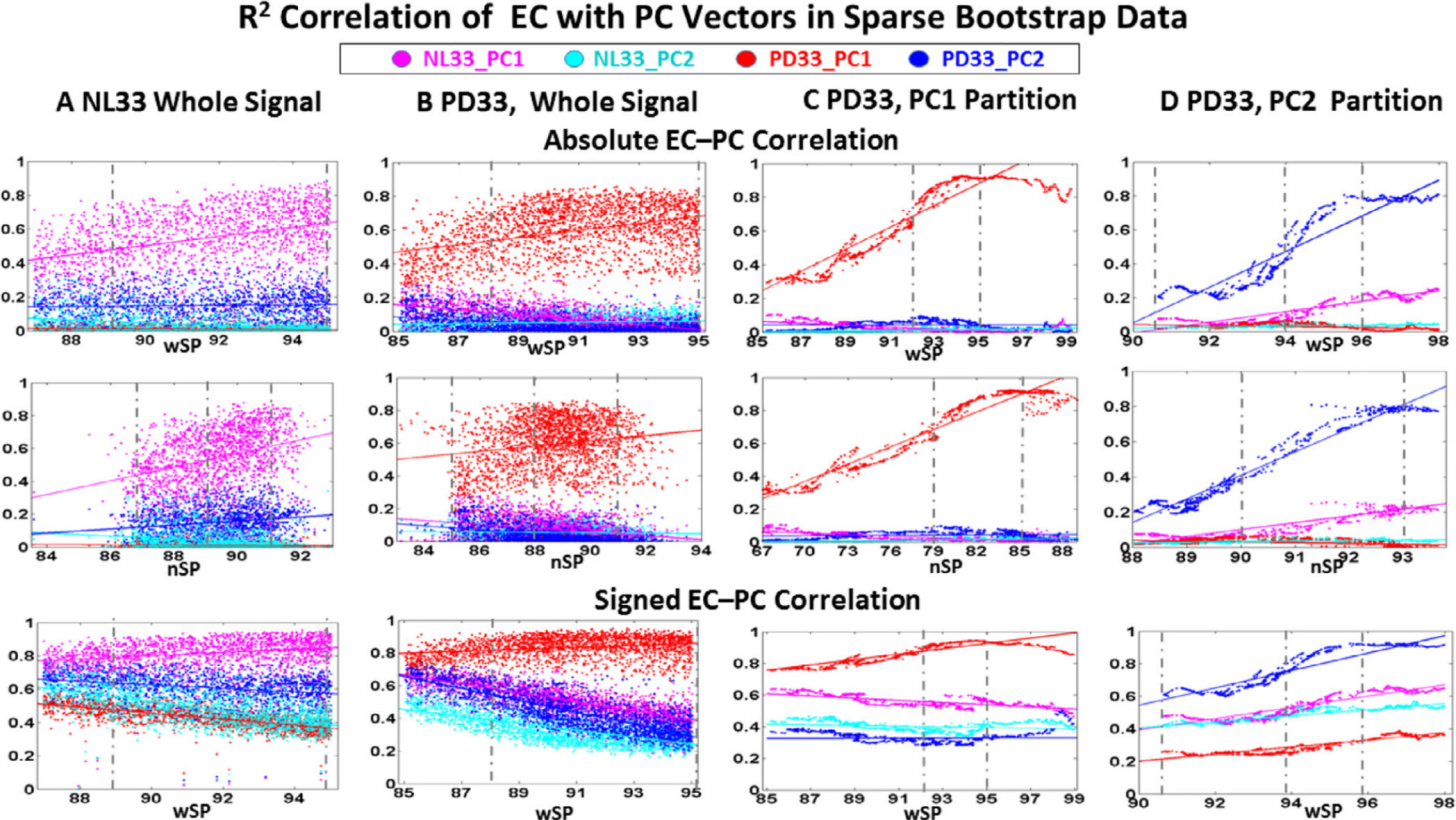
Pearson’s correlation of EC with PC region weights in bootstrap samples. *Top:* The top two rows represent r^2^ correlation between the EC of each data set with the absolute region weights of the healthy and disease PCs plotted against whole brain sparsity (*1st row*) and connected network sparsity (*2nd row*). *Bottom:* The last row is the r^2^ correlation of the signed PC weight with the EC vector signed similarly to the PC vector and plotted against whole brain sparsity. Colors correspond to the assigned color of the PC in the legend for which EC correlation is evaluated (red for PD33_PC1, blue for PD33_PC2, magenta for NL33_PC1 and cyan for NL33_PC2). Dashed vertical lines represent visible limits for the range of lower and higher sparsity values for which the correlation was considered to be stable and was used in further analysis of the most robust connections. **A:** NL33 whole brain data; samples 200, 1730 cases, **B:** PD33 whole brain data; samples 200, 2319 cases, **C:** PD33_PC1 partition data; samples 500, 2015 cases, **D:** PD33_PC2 partition data; samples 400, 1932 cases.

The NL33 whole brain and the PD33 whole brain and PD33 PC partition EC vector weights determined in bootstrap data were evaluated for their Pearson’s r^2^ correlation with each of the predetermined NL33 and PD33 PC1 and PC2 vector weights. These values are illustrated in Fig. 5 as a function of whole brain sparsity (wSP, *top* and *bottom rows*) and connected network sparsity (nSP, *middle row*). The top and middle rows are evaluated for correlation of the derived positive EC with absolute PC weights and the bottom row is for the simulated similarly signed EC with the original signed PC weights. For over 200 samples in each group whole brain data, and over 400 samples in PC partition data, values for all 95 regions are input into the GLASSO algorithm and evaluated for incremental values of the penalty parameter resulting in approximately 2000 cases, signified by individual points on each graph. Results are tabulated for whole brain sparsity wSP within the overall range of 85 to 99%. For all cases, EC is evaluated for the connected subnetwork determined in a data driven way to have a variable number of regions less or equal to 95. Thus, the corresponding connected network sparsity nSP is typically less than wSP (*middle row*) for the same number of connected edges. The disparity is greatest for the PD33 PC1 and PC2 subnets (Fig. 5C, D) that were determined by the algorithm to be fully connected for far fewer than 95 nodes, whereas connected networks in whole brain data (Fig. 5A, B) involved most if not all of the 95 nodes. However, the derivation PC vectors correspond to signed weighted values for all of the 95 nodes. Thus, correlations with the EC vectors of the subnets is evaluated for the reduced number of regions in the subnet space. In whole brain networks, additional regional connections may be contributed by separate networks, which can result in discontinuities in correlations at the limits of the connected network sparsity range. It is evident from Fig. 5C, D that correlations with the PC from whose partition space the EC vector was derived is higher than for other PCs and higher than whole brain EC/PC correlation illustrating the direct correspondence of the graphical subnet and derivation PC pattern map representations.

The graphs of EC correlation to PC region weight in whole brain NL33 and PD33 data (Fig. 5A, B) illustrate that sparse connectivity was dominated by connections of the primary PC of the preliminary PCA data analysis in our data. However, to a variable degree, the regional network associations of secondary underlying PC networks are also reflected (because of their actual intrinsic connectivity), as well as potential correlations with independent networks. As sparsity increases, the correspondence to the prominent PC increases as weaker connections are removed while that to the less prominent secondary PC generally decreases. Correlation of the EC in one data set to the PCs of an independent data set illustrates the degree of similarity of the corresponding networks within their common nodal subspace. Thus, for the whole brain healthy data (Fig. 5A), the correlation is high for the NL33_PC1 network shown in magenta and low for NL33_PC2 (cyan) while the prominent correlation of the disease group PD33 whole brain EC is highest to PD33_PC1 shown in red and low for PD33_PC2 shown in blue in Fig. 5B. The correlation to the PD33_PC1 disease network is the lowest in the healthy whole brain data of Fig. 5A shown in red, while correlation with PD33_PC2 (blue) is closer to the normal correlation points. This suggests that the primary disease pattern is an abnormal pattern while the secondary PC of the disease data is fundamentally normal. A further, indication of near normal expression of PD33_PC2 is that in the whole brain disease data (Fig. 5B), blue points corresponding to the PD33_PC2 network are interspersed within the range of the healthy NL33_PC1 (magenta points). These associations are more evident in the bottom row of Fig. 5 where PC–EC correlations are higher for EC vector weights signed similarly to those of the PC weights.

The correspondence of the sparse graph EC of the partition data to the preliminary PC derivation weights is more clearly defined in the graphs of Fig. 5C for PD3_PC1 and Fig. 5D for PD33_PC2. Thus, red points dominate in Fig. 5C for the correlation of EC with PC1 in the PD33_PC1 partition, and blue points dominate in Fig. 5D for the correlation of EC with PC2 in the PD33_PC2 partition. Here we can detect a sub range of high sparsity values (noted by vertical dashed lines) for which the EC signal correlation is higher and more stable. For the PD33_PC1 disease network partition (Fig. 5C), correlation of EC with the secondary and normal PCs is minimal (diverging of red from other colored points) indicating the singularity of this pathway, while there is some correlation with the normal NL33_PC1 network evident for signed correlation (Fig. 5C, *bottom*). The reduced PD33_PC1 subnet space includes some highly weighted nodes of the NL33_PC1 95 region vector though not necessarily of the same polarity (Fig. 1B, E). As noted above, the negative correlation of the original NL33_PC1 and PD33_PC1 patterns raises the possibility that the disease pattern develops, at least in part, from the normal topography. However, this simulated bootstrap correlation is reduced at high sparsity due to the increased influence of non-correlating highly weighted PD33_PC1 nodes. For the PD33_PC2 partition (Fig. 5D), correlation with PC2 is high but not as pronounced (blue points), while correlation with the healthy NL33_PC1 network (magenta points) is evident even for absolute values, indicating that this network is closely related to the healthy group network and differs greatly from the disease network.

### 3.5. Significant connections in bootstrap data

**Fig. 6.**
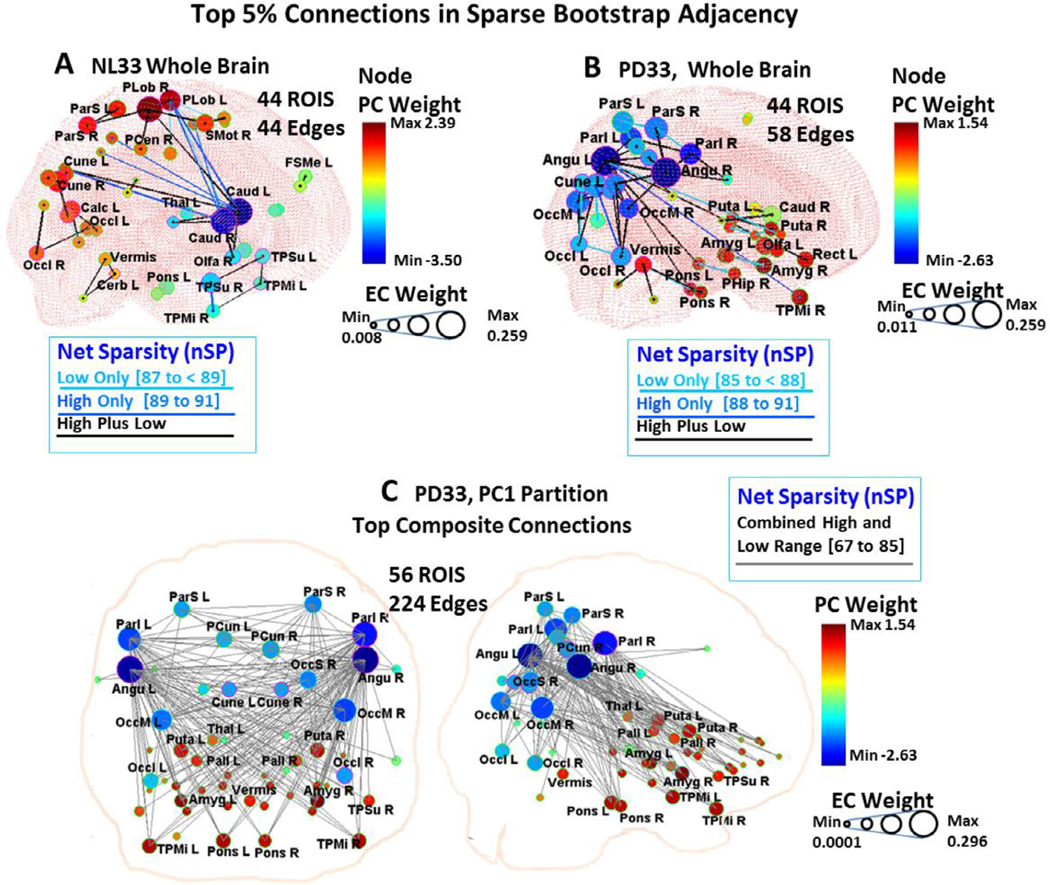

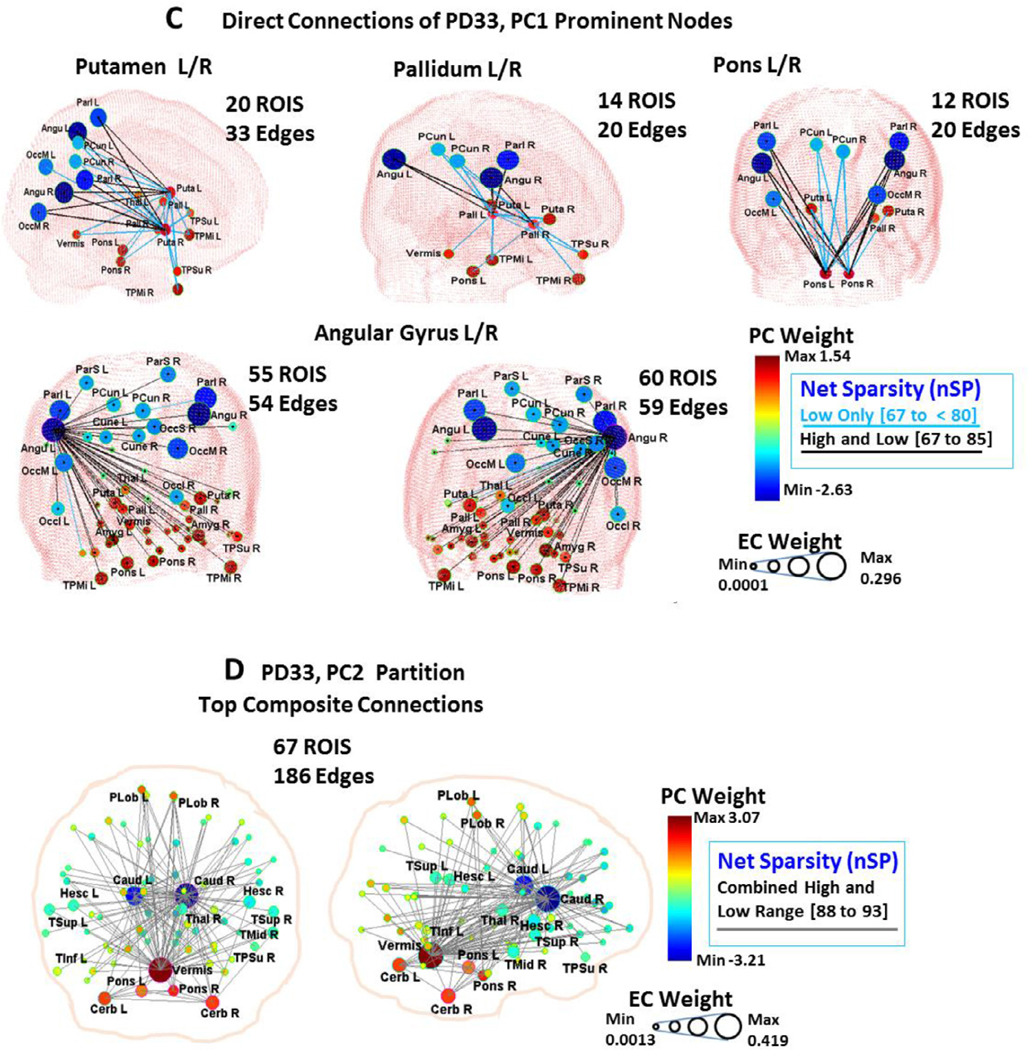
Top connections of composite bootstrap adjacency matrices. 3D displays (***A to D***) are depicted for composite bootstrap connections of whole brain healthy NL33 (***A***) and PD33 (***B***) data and for PD33 partitions PC1(***C***) and PC2 (***D***) for 95 ROIs. Corresponding circular displays are shown in Fig. S6. A dual sparsity range of the adjacency matrices was used to depict the top 5% of connections (95% of bootstrap cases) at lower sparsity and those that are also maintained at high sparsity ranges in each group. Note, however, that the high sparsity connections (dark blue and black or gray) were also present in the top 5% of the lower sparsity range cases (light blue and black) for the partition data and for most of the whole brain data. Connections that are evident only at lower sparsity (light blue lines) are depicted in supplemental circular displays and in the 3D displays of the whole brain data, and for connections of selected nodes in the PD33_PC1 partition (***C****, bottom 2 rows*: left and right angular gyrus, putamen, pallidum and pons). The diameter of the nodes reflects mean EC weight of the component adjacency matrices; the color of the nodes corresponds to the signed weight of the partition PC of that correlated best with the EC vectors for whole brain data: **A:** NL33 Whole Signal 200 samples, Total 1730 cases, 44 edges, node color: NL33_PC1; Lower range wSP: 87% to < 95%, nSP: 87% to < 89%, 618 cases, Top 5%: (37 edges, light blue and black for 29 edges also included in the higher sparsity range); High range wSP: 89% to 95%, nSP: 89% to 91%, 838 cases, Top 5%: (36 edges, blue and black for 29 edges also included in the lower sparsity range). **B:** PD33, Whole Signal 200 samples, Total 2319 cases, 58 edges, node color: PD33_PC1; Lower range wSP: 85% to 95%, nSP: 85% to < 88%, 723 cases, Top 5%: (56 edges, light blue and black for 38 edges also included in the higher sparsity range); High range wSP: 88% to 95%, nSP: 88% to 91%, 1396 cases, Top 5%: (40 edges, dark blue and black for edges also included in the lower sparsity range). **C:** PD33_PC1 Partition, 500 samples, Total 2015 cases, node color: PD33_PC1; Composite 3D display: Combined range wSP: 85% to 95%, nSp 67% to 85% 1493 cases, Top 5% (224 edges, gray); Circular display (Fig. S6 C): Lower range wSP: 85% to < 92%, nSP: 67% to < 80%, 535 cases, Top 5% (312 edges, light blue and black for 224 edges also included in the higher sparsity range); High range wSP: 92% to 95%, nSP: 80% to 85%, 958 cases, Top 5%: (224 edges, black, shown gray in composite display). **D:** PD33_PC2 Partition, 400 samples, 1932 cases, node color: PD33_PC2; Composite 3D display: Combined range wSP: 90% to 96%, nSp 88% to 93% 478 cases, Top 5% (186 edges, gray); Circular display (Fig. S6 D): Lower range wSP: 90% to < 94%, nSP: 88% to 90%, 788 cases, Top 5% (275 edges, light blue and black for edges also included in the higher sparsity range); High range wSP: 94% to 96%, nSP: 90% to 93%, 203 cases, Top 5% (186 edges, black; shown gray in composite display).

Composite 3D connectivity configurations of significant edges between the 95 ROI nodes found in bootstrap analysis are depicted in Fig. 6 for the whole brain healthy NL33 (Fig. 6A) and PD33 (Fig. 6B) data and for PD33_PC1 (Fig. 6C) and PD33_PC2 (Fig. 6D) partitions. Corresponding circular displays are presented in Fig. S6. For these displays, cases were evaluated for samples within a range of penalty values that exhibited stable values of EC to PC correlation for higher and lower sparsity ranges (depicted by vertical demarcation lines in Fig. 5 for the corresponding sparsity ranges). For whole brain NL33 and PD33 data, the high and low ranges (specified in Fig. 6 legend) were determined from the connected sparsity range (nSP) to exclude the unreliable low and high limit region data evident in Fig. 5A, B (*center*). This resulted in an overlap in high wSP ranges 87% to 95% for NL33 and 88% to 95% for PD33, with corresponding lower wSP ranges that were attributed to multi-network influences in the whole brain data. For specific network partition data, there was a reduced, more uniform connected sparsity range and non-overlapping correspondence between whole brain and subnet sparsity values. For the PD33_PC1 partition the high sparsity range was 92% to 95% wSP and for the PD33_PC2 partition it was 94% to 96%. The corresponding number of cases within each high sparsity range was 838 for NL33 whole brain, 1396 for PD33 whole brain, 958 for the PD33_PC1 partition and 203 for the PD33_PC2 partition. For lower sparsity, the corresponding number of cases was 618, 723, 535 and 788, respectively.

In the displays (Fig. 6) for the 3D configurations of whole brain and partition data, the top 5% most probable connections (95% or more of bootstrap cases) at high sparsity are shown in dark blue or black (detailed in the legend). The high sparsity prominent edges were also present in the lower sparsity top 5% adjacency matrices for partition data. For whole brain data most high sparsity edges were also present at lower sparsity and are depicted in black. Additional edges for the upper 5% of connections in a lower sparsity range that are not also present at higher sparsity are depicted in lighter blue colors on circular (Fig. S6) and 3D whole brain displays (Fig. 6A, B) and in individual partition nodal pathways (Figs. 6C and S6, *bottom*). For the 3D displays, the diameter of the nodes reflects mean EC values of the corresponding sample cases and the color of the nodes reflects the weight of the highest correlated PC vector.

#### 3.5.1. Whole brain significant connections in bootstrap data

In contrast to the NL33 whole brain strongly connected configuration (95 regions, 599 edges) displayed in Fig. 2 (*top left*) for the original sample, only 36 edges depicted in the composite bootstrap display (Fig. 6A) were present in 95% or more of 838 high sparsity cases (blue/black), with an additional 8 edges present only at lower sparsity (light blue) for a total of 44 edges in over 95% of lower and higher sparsity cases. The relatively small number of common edges (total 44 compared to 4465 possible connections) over the range of samples may stem from the extensive networks that are active in the healthy brain varying in each sample. The graphs formed by the prevailing connections in this figure were not fully connected, representing elements of NL33_PC1 and NL33_PC2, as depicted in Fig. 2 (*middle and bottom left*). The caudate nuclei were dominant nodes in the NL33 whole brain graph (Fig. 6A), with connections to the thalamus, paracentral lobule, and temporal areas as in NL33_PC1 (Fig. 2, *middle left*). Also, as in NL33_PC2, this graph contained separate connections linking frontal, parietal, and occipital regions, and motor areas (Fig. 2, *bottom left*). Connections of the pons, vermis and cerebellum were visible in both NL33_PC1 and NL33_PC2, suggesting a dual role in whole brain functionality: connecting to the caudate, putamen, globus pallidus, and paracentral areas in NL33_PC1, and to frontal and motor areas in NL33_PC2. Of note, these regions appear detached in the whole brain bootstrap configuration (Fig. 6A).

The whole brain PD33 disease network (Fig. 2, *top right*) of the original sample at maximum sparsity was fully connected for a much smaller number of edges (95 regions, 483 edges) than in the healthy group in accordance with previous findings of decreased characteristic path length, increased clustering and increased small-world properties in PD networks (Ko et al., 2018). A major reconfiguration of the healthy topography of Fig. 6A is evident in the disease topography of Fig. 6B for 58 common top connections in whole brain bootstrap assessment. Paracentral, supplemental motor and frontal area nodes are notably diminished and the centrality of the caudate regions is reduced. Here, predominant connections involve links of the angular gyrus to the inferior parietal and occipital association regions, and to the precuneus, temporal cortex, and pons — regions relevant to PD33_PC1 (Fig. 6C). A strong connection is observed between the right and left putamen and between right and left pallidum at lower sparsity; connections between these and other regions are not prevalent in whole brain bootstrap samples.

#### 3.5.2. Significant connections in subnet partition bootstrap data

A clearer picture of disease relevant connections is obtained from the illustrations of the robust edges of the PD33_PC1 partition data (Fig. 6C) derived in the joint lower sparsity wSP range (85% to less than 92%), (67% to 80% nSP) indicated by light blue and black edges, and the high sparsity wSP range (92% to 95%), (80% to 85% nSP) indicated by dark blue or black edges. The main features of the configuration of the original sample (Fig. 2, *right center*) that involved 347 edges are preserved in the 224 edges present in over 95% of the composite 958 bootstrap cases at high sparsity (Fig. 6C, *top row*). The individual pathways in the joint sparsity range of the angular gyri, putamen, pallidum and pons depicted in Fig. 6C (*middle and bottom rows*) illustrate the salient functional connections of these prominent PD nodes to all of the PD relevant nodes of the PD33_PC1 partition. The angular gyri (Fig. 6C, *bottom row*) function as hub nodes (see also Table S2) fully connecting to all of the nodes within the partition even at high sparsity. The interconnections between positive PD33_PC1 regions of the basal ganglia, pons, vermis, amygdala and temporal areas (Fig. 6C, *middle row*) are only apparent at lower sparsity indicated by a lighter blue color.

The composite PD33_PC1 partition adjacency matrix for the top 5% bootstrap connections (present in over 95% of the corresponding sample bootstrap adjacency matrices) is shown in Fig. S7 for the lower (85% to less than 92%) sparsity range (*left*) and the higher (92% to 95%) sparsity range. The matrices are similar to that of Fig. 4 (*left*) with a notable absence of high EC positive to positive edges (red points) at high sparsity. Edges between red nodes are apparently more sensitive than positive to negative connections, and are suppressed by the increased penalty imposed in GLASSO at high sparsity. Among the strongest of these connections between red nodes, were left and right putamen, pallidum and amygdala (Fig. S7, *left* and Fig. 6C, *middle*). The composite bootstrap adjacency at high sparsity (equivalent to that for the combined range configuration of Fig. 6C, *top*) consists of 224 edges of a connected network between 56 ROI nodes with a corresponding EC vector (bEC1). The vector map representation of the signed EC (sbEC1) is presented in Fig. S8 and is highly similar to that of the original sample (Fig. 3, *upper right*). The Pearson’s correlation of the sbEC1 vector weights with the PD33_PC1 vector weights was *r* = 0.97, *p* < 0.001. Subject scores evaluated for the sbEC1 vector pattern were found to be highly discriminative of prospective early PD13 patients from those of healthy NL13 subjects (*p* = 0.0008, AUC = 0.88, Fig. S8, *bottom right*).

The PD33_PC2 partition pattern exhibits features that are closer to normal (see text), with major connections indicated by the composite bootstrap pattern (Fig. 6D) centered between the caudate and vermis to supplementary motor areas including broad connectivity to frontal, parietal, temporal and cerebellar areas. Connections between the caudate, pons, cerebellar hemispheres and vermis, thalamus, and paracentral cortex and supplementary motor areas are maintained in both the NL33_PC1 (Fig. 2, *middle left*) and PD33_PC2 (Fig. 2, *bottom right*; Fig. 6D) partition networks. Interestingly, connections to the basal ganglia are visible only in the healthy NL33_PC1 network, suggesting that in disease, these normal pathways become part of the abnormal PD33_PC1 topography.

### 3.6. Summary of findings

(1) In accordance with our proposed methodology, an initial regional PCA of our PD33 and NL33 data group was performed that determined PD33_PC1 as the principal component whose expression scores best discriminated patient groups from healthy controls (Fig. 1A–J). The high correlation (*r* = 0.79, *p* < 0.001) of the regional PD33-PC1 (disease) pattern to the voxel-based pattern PD33_PC1 was noted in Fig. 1A, B. This voxel pattern, derived solely from disease group data, correlates highly with the well-known voxel based disease pattern PDRP (*r* = 0.89, *p* < 0.001) derived from the joint PD33 and NL33 groups (Ma et al., 2007; Spetsieris et al., 2015).
(2) To determine the major underlying connections of the 95 regional NL33 and PD33 data samples, whole brain GLASSO/SICE analysis was performed to obtain the sparsest adjacency matrices that insured a fully connected network in each data set (Fig. 2, *top, left* and *right*). Sparse representations were also determined for the PD33_PC1 and PC2 partition data (Fig. 2, *lower, right*) and the NL33_PC1 and PC2 (Fig. 2, *lower, left*).
(3) The correlation of the PD33 whole and partition signal graph adjacency EC vectors of the GLASSO/SICE analysis with that of the values of the PD33_PC1 vector (Fig. 3) are represented for EC region weights signed similarly to the PC region weights. High correlation between absolute PC, EC vector values and even higher correlation between signed values indicate the strong association between the connectivity of the graphical representations to that of the corresponding PC representations.
(4) The Laplacian Fiedler vector nodal order of magnitude was used to re-order the adjacency matrices to illustrate the modular associations and hub network connections in the PD33 whole brain and PC partition networks (Fig. 4).
(5) We performed bootstrap analysis to validate our original findings and determine the most robust connections. The Pearson’s correlations of the absolute and signed PC vectors with the absolute and similarly signed EC vectors of the whole brain and partition graph networks was evaluated in extensive bootstrap iterations over a wide range of sparsity levels (Fig. 5) to determine similarity across networks and stability across sparsity ranges.
(6) The prominent connections that were present in over 95% of the bootstrap samples of each data set were assessed and depicted in 3D nodal brain and 2D circular configurations (Figs. 6 and S6A–D). The nodal connectivity pathways within the composite PD33_PC1 bootstrap data were traced for major network nodes including the putamen and pallidum, pons and angular gyri (Fig. 6C, *middle and bottom*).
(7) The discriminant potential of the signed EC vectors of the original PD33 whole brain and PC partition data was evaluated in independent early PD patients and matched healthy controls (Fig. 3) and by comparison in high sparsity PD33_PC1 bootstrap data (Fig. S8).

## 4. Discussion

In this study, our objective is to present a new approach using graph theoretical inverse covariance estimation (GLASSO/SICE) to delineate the implicit network connections between regions associated with PCA-derived spatial covariance patterns in the same data. By estimating the matrix of partial correlations, GLASSO/SICE gives a read-out of the relevant connections without nodal “third party” interactions. We employ this methodology to elucidate the pathological network associations underlying the PDRP metabolic topography, which is considered to be a characteristic feature of the disease. Thus, we observed wide-spread strong cross-coupling of the low weighted (negative) metabolic regions of the angular gyri and inferior parietal regions acting as external hubs linked to extensive areas, including the more metabolically active (positive) local regions of the putamen and pallidum. More sensitive interactions were detected only at lower sparsity levels between positive regions of the basal ganglia, pons and vermis acting as local hubs. Frontal connectivity was noticeably suppressed in the disease component. In contrast, healthier interactions involved the caudate with cerebellar and motor regions acting as inter-linked hubs within the second principal component.

The PDRP model may be effective because of the broad range of clinical manifestations expressed by the PD33 patients (Eidelberg, 2009; Ma et al., 2007), which enables shared underlying disease features to be clearly distinguished — even in the sparse state of the whole brain data. Considering that graph theoretical analysis is performed on data from a single group of similar subjects, we used as the disease proxy the closely associated regional PD33_PC1 pattern derived in the PD33 patient group alone. In this data-driven approach, the PDRP pattern itself was not directly involved in the analysis. In addition, for comparison, we similarly examined the healthy NL33 group data that was used in the original derivation of the PDRP topography.

Whole brain GLASSO analysis of the data was performed to illustrate that the resulting sparse configuration represents dominant connections from all of the underlying networks, which do not necessarily reflect the disease process alone, particularly in early stage patients. Thus, although prominent connections are visible in the whole brain sparse composite connectivity configurations (Fig. 6A, B), a single coherent fully connected network was not evident because these representations are composites of different combinations of multiple underlying subnetworks that may be functionally active within each brain. Instead, we applied the GLASSO methodology to specific PC partitions of the data signal in order to map disease specific network associations. This approach differs from other GLASSO-based sparse PCA methodologies (Zou et al., 2006) that derive sparse PCs directly in whole data. Rather, here the original PC disease partition data is evaluated for all 95 regions and is subsequently reduced according to the GLASSO penalty function. Thus, sparse partition networks are not pre-identified as this may reduce the robustness of the disease-specific signal. However, using, specific subnetwork PC partitions of the data, we obtain a clearer picture of the role of prominent regional associations in each of the major component subnetworks.

The primary healthy NL33_PC1 network (Figs. 1E and 2, *middle left*) indicates connections of the caudate to the rest of the basal ganglia, and to the thalamus, cerebellum, and pons, as well as paracentral and temporal cortical regions, representing normal sensorimotor pathways. In addition, in health, connections are evident in a separate NL33_PC2 pathway (Figs. 1F and 2, *bottom left*) linking medial frontal and supplementary motor areas to the posterior cingulum and medial parietal and occipital regions, as well as the pons and cerebellum — overlapping considerably with the metabolic default mode network (Spetsieris, 2015).

By contrast, the PD33_PC1 disease network (Figs. 1B, 2, *middle right*, and 6C) exhibits distinct functional connections linking negatively weighted areas of the angular gyri, parietal and occipital regions to positive activity in the putamen and pallidum as well as the pons, vermis, amygdala and temporal lobe. Salient connections between the pons and the basal ganglia appear to be suppressed at high sparsity (Fig. 6C, *middle right*) but are strongly linked to the highly negative parietal and occipital regions, which contrasts with the healthy connections observed in NL33_PC1 (Fig. 2, *middle left*). In addition, the PD33_PC1 representations exhibit a striking absence of connectivity to frontal regions and a suppression of connections linking the caudate with the paracentral lobule and supplementary motor cortex. In contrast, PD33_PC2 (Figs. 1C, 2, *bottom right*, 6D) involves strong functional interactions between the negative caudate and positive vermis, cerebellar hemispheres, and pons, which are similarly connected with nodes in the frontal cortex and with paracentral, supplementary motor, temporal, and parietal regions. This network exhibited high EC–PC correlations (Figs. 5D and 1C) with the healthy network configuration of NL33_PC1 (Figs. 1E and 2, *middle left*). Notably the PD33_PC1 network also exhibited high signed EC r^2^ correlation (Figs. 5C and 1B) with the healthy network of NL33_PC1 but the inherent polarity of these correlations cannot be inferred from these graphs alone. We can assume that this correlation is innately negative based on the PC correlation (*r* = −0.58, *p* < 0.001) of the original associated topographies (Fig. 1B and E) as discussed in the text. This suggests that in PD data, this source of normal (or near normal) metabolic activity is segregated from the disease-related PD33_PC1 network (Spetsieris et al., 2015). This is also indicated by the difference in prospective discriminative scores between PD33_PC1 and PD33_PC2 (Fig. 1, *bottom left*). Notably, changes in cerebellar function have been associated with cognitive decline in PD patients (Blum et al., 2018; Mattis et al., 2016; Schindlbeck and Eidelberg, 2018), which occur at a much slower pace than motor-related abnormalities, and changes in caudate–frontal pathways have also been implicated in this process (Carbon et al., 2003). The separate PD cognitive pattern (PDCP) identified by Huang et al. (2008, 2007) was further shown by Mattis et al. (2016) to be spatially and functionally distinct from that seen in relation to AD. Significant correlations have been demonstrated between caudate dopaminergic innervation and PDCP expression values (Holtbernd et al., 2015; Niethammer et al., 2013). In addition, regions of the caudate and cerebellum as well as motor and parietal regions of networks derived in activation data were previously shown to be associated with learning performance (Carbon et al., 2003). The whole brain sparse configuration of the PD33 adjacency connections (Fig. 6B) exhibits elements of both of the PD33_PC1 and PD33_PC2 connections in independent disjoint clusters but primarily exhibits those associated with the first PC. The separation of basal ganglia connections of the putamen and globus pallidus on one hand (exemplified by PD33_PC1), and the caudate on the other (exemplified by PD33_PC2) is consistent with the inherent segregation of these circuits in the healthy brain (Alexander et al., 1986; DeLong and Wichmann, 2007). That said, the current data indicate a functional dissociation of the corresponding networks in the context of disease, with preferential involvement of connectivity in motor pathways shedding light on the basis of these separate networks in PD (Martinu and Monchi, 2013).

The functionality of the angular gyrus in PD has not been fully investigated. It is evident from the finding in this study that the angular gyrus is centrally and integrally connected with all component regions of the disease network as well as with low centrality regions that are not directly connected with core regions in the striatum and brainstem. Previous studies in healthy subjects have attested to the multi-functionality of the angular gyrus and in its integrating role in spatial cognition (Sack, 2009; Seghier, 2013). Non-invasive techniques such as transcranial direct current stimulation (tDCS) have demonstrated that external stimulation of parietal areas including the angular gyrus can induce extensive changes in resting-state activity of sensorimotor and cognitive areas (Clemens et al., 2014). However, for PD patients, applications of tDCS have been limited to stimulation of motor and frontal areas with modest benefit (Costa-Ribeiro et al., 2016; Dobbs et al., 2018; Ganguly et al., 2020). Knowledge of the specific PD circuitry illustrated in this study may guide future external modulation of key regions using newer therapeutic methodologies.

We are currently using the techniques described here to investigate the modulation of functional connectivity in disease specific PCs in a cross-sectional study of PD patients with disease of varying duration.

### 4.1. Limitations

This study attempted to capture the signature functional connections that characterize PD within the wide range of disease duration characterized by the PD33 group. Separate studies are needed to address connectivity differences relating to specific clinical indices such as disease duration and motor disability ratings.

The accuracy of the PD33_PC1 representation of regional functional connectivity as a proxy to PDRP voxel-based connectivity is subject to scaling and regional parcellation effects (Fornito et al., 2010; Sporns, 2013). The PDRP reflects variance in the full brain voxel space of combined patient and control groups, whereas the PD33_PC1 is derived from variance in 95 regional mean values within the disease group alone. Further effects from individual sub-regional partitions such as that of the putamen or cerebellum that may have specific functional significance have not been addressed. Moreover, the current study focused on the first two PC patterns although additional topographies may capture other important network functions in PD or in the normal brain.

## Supplementary Material

1

## Abbreviations:

wSP: whole brain network sparsity
nSP: connected network sparsity
subnet: connected subnetwork within a PC partition space
AAL region names: Table S1
