# Supplementary material for: Spectral guided sparse inverse covariance estimation of metabolic networks in Parkinson’s disease
Source: Neuroimage. Author manuscript; Available in PMC 2021 Sep 1. (PMC8409106; doi:10.1016/j.neuroimage.2020.117568)
Supplement: 1 [file NIHMS1668477-supplement-1.docx]

**Supplementary Figures and Tables**

**
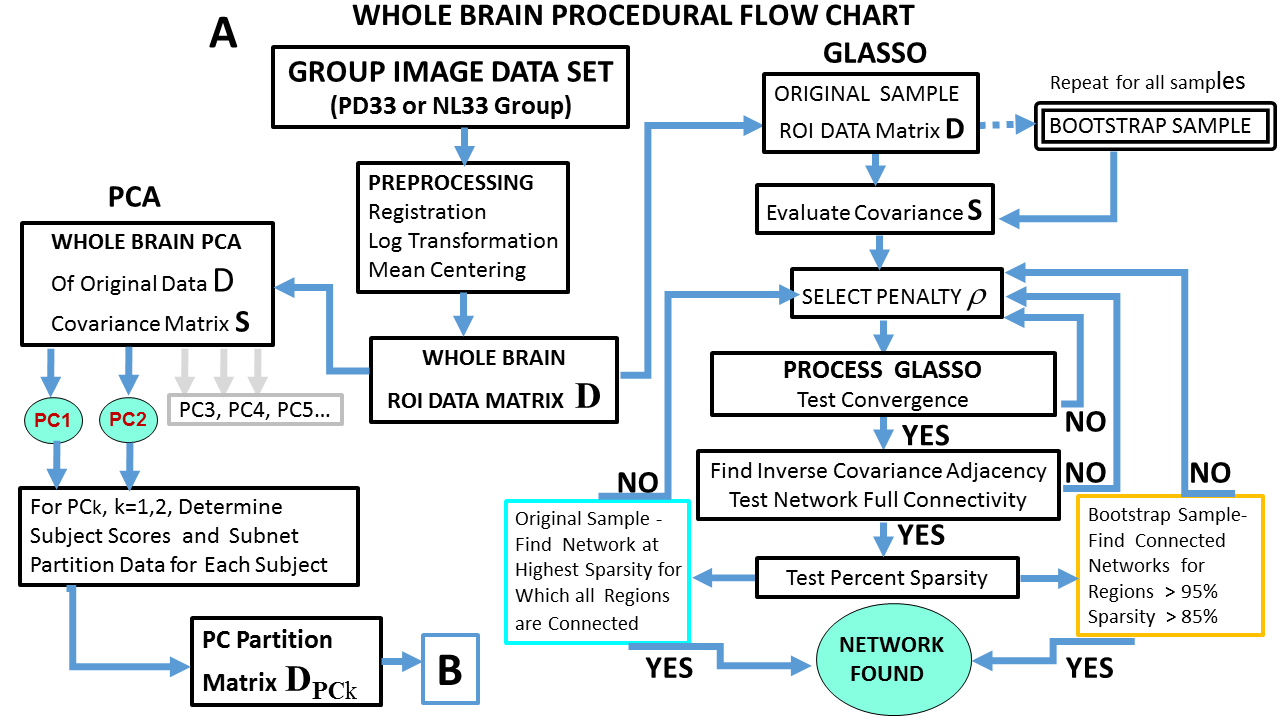
**

**
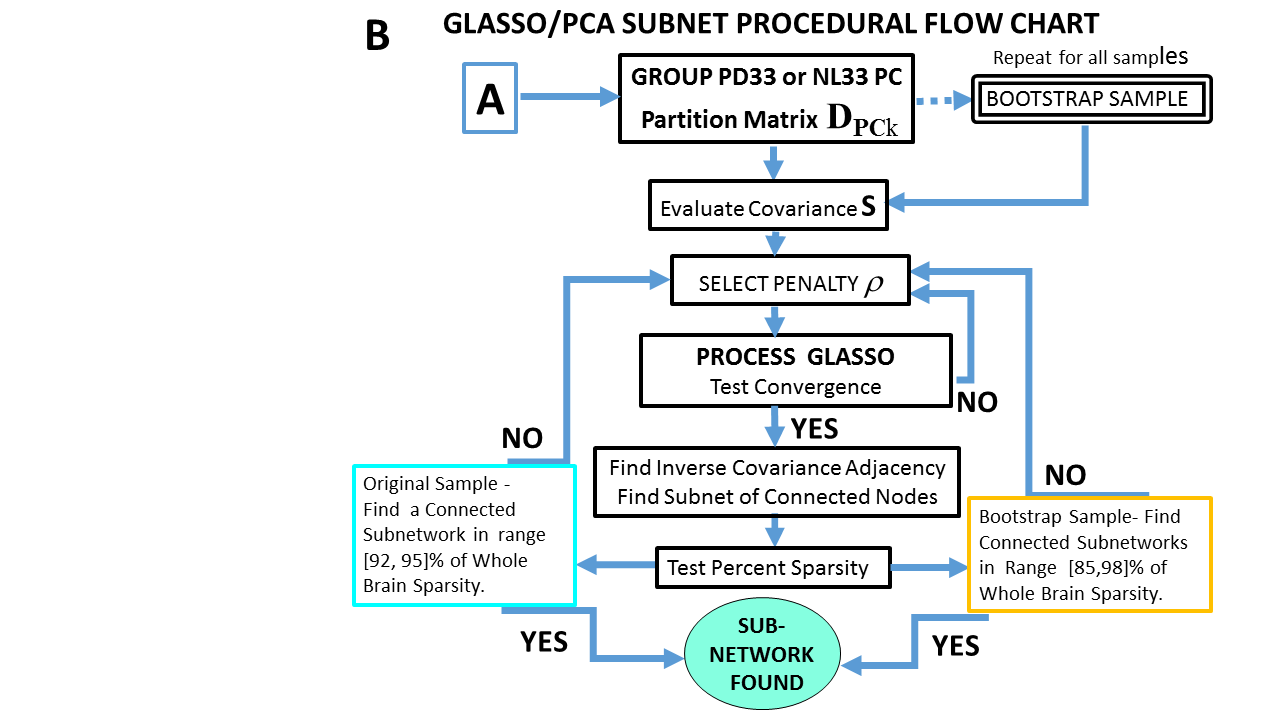
**

**Fig. S1. GLASSO/PCA procedural flow chart.** Whole brain data is initialized in accordance with the SSM protocol of registration, log transformation and centering, resulting in the subject by region group data matrix **D**. The regional covariance matrix of the data matrix **D** is evaluated and processed separately using PCA (**A***, left*) and using GLASSO (**A***, right*). The whole brain PCA process (**A**, *left*) is used to derive significant PCs and corresponding partition data **D_PC_**_k_ for the subnet analysis **B**. The whole brain GLASSO process (**A**, *right*) is performed on the covariance matrix of the data matrix **D** to estimate sparsified inverse covariance matrices and associated adjacency matrices for the original data **D** and bootstrap samples of **D** for a range of penalty values. Results are tabulated for fully connected, high sparsity (wSP> 85%) networks involving all (95) or over 95% (90) nodes in bootstrap cases. The GLASSO subnet derivation procedure B is performed on the covariance matrix of the partition data **D_PC_**_k_ involving all (95) regions to estimate the sparsified inverse covariance matrices and associated adjacency matrices of the partition data **D_PC_**_k_ and bootstrap samples of **D_PC_**_k_ for a range of penalty values. Results are tabulated for the fully connected subnetworks that typically involve fewer than 95 nodes, for high whole brain sparsity in the range [85 to 98]%.

**
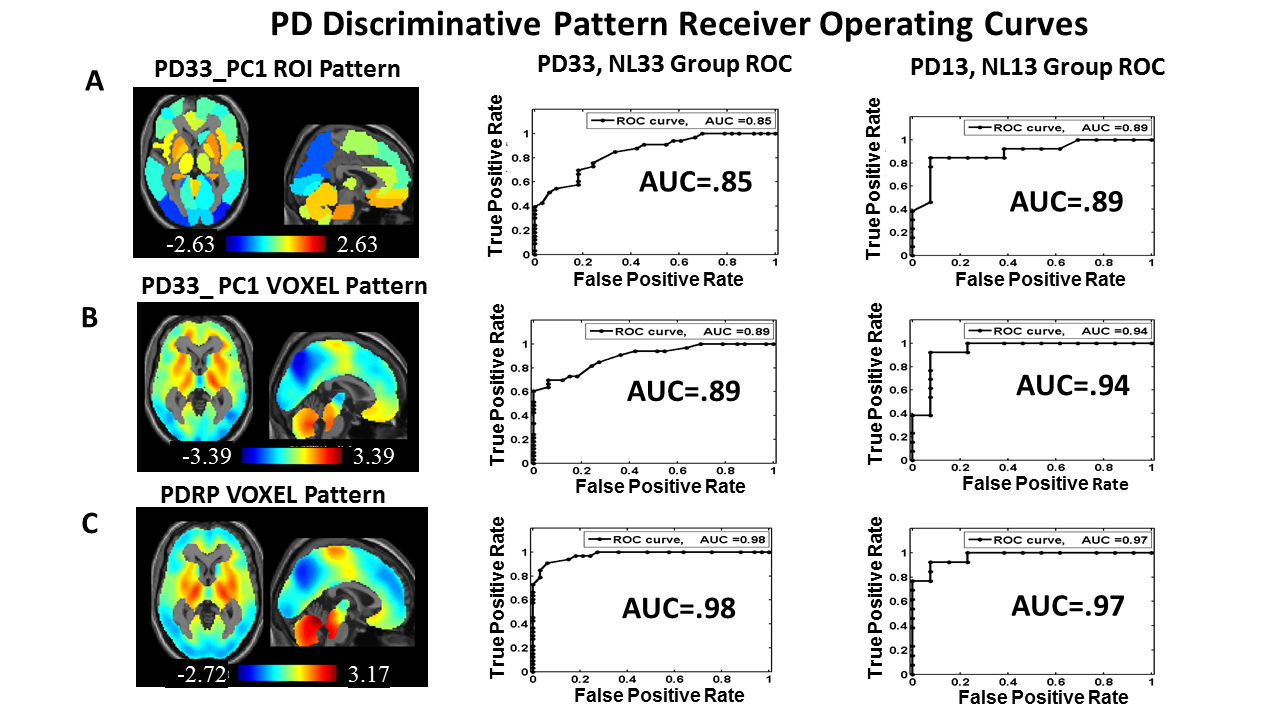
**

**Fig. S2. PD discriminative pattern receiver operating curves.** For each of the disease patterns, the PD33_PC1, ROI pattern vector map (**A**), the PD33_PC1, voxel pattern (**B**) and the PDRP, voxel pattern (**C**), ROC curves are shown for both the PD33, NL33 group score data (*middle column*) and the prospective PD13, NL13 group score data (*right column*).


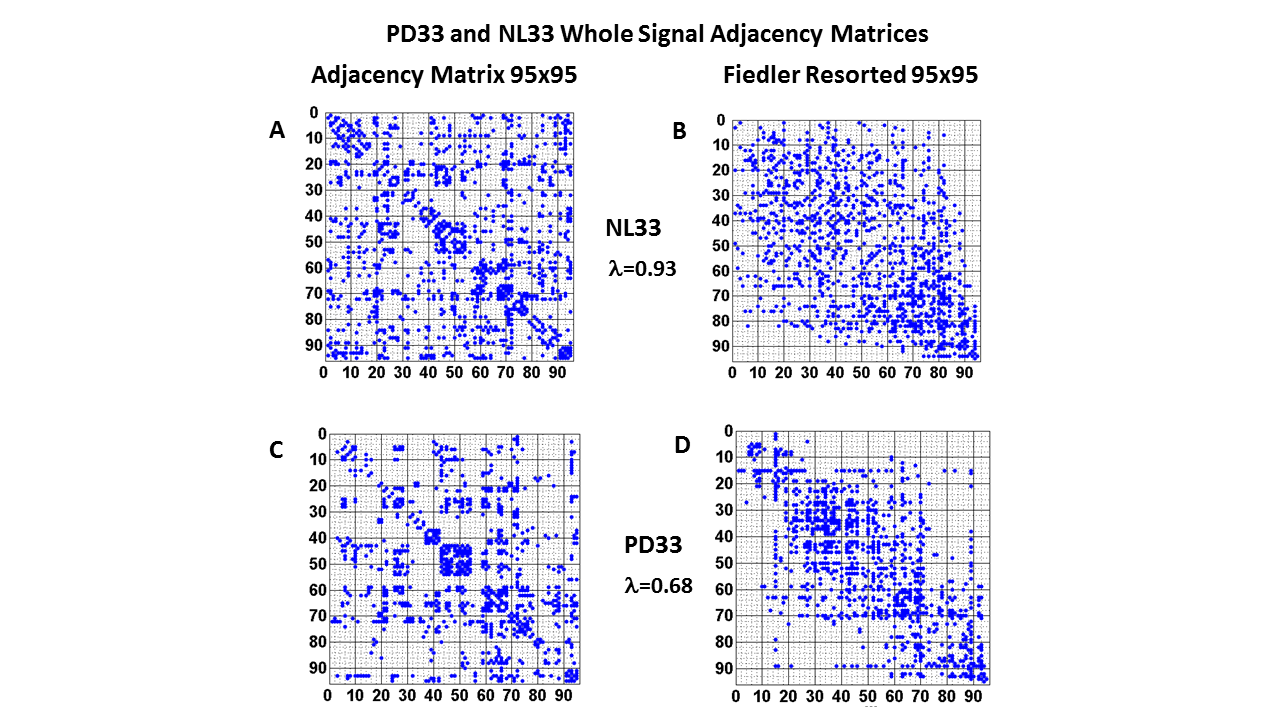


**Fig. S3. Sparse network whole brain adjacency matrices.** Whole brain GLASSO derived adjacency matrix of fully connected network at maximum sparsity of the original group samples NL33 (*top*) and PD33 (*bottom*). The axes are numbered in line with the nodal atlas index (*left*) or resorted in accordance with their nodal magnitude in the corresponding Fiedler vector (*right*) of the graph Laplacian matrix. Each point represents an edge connecting a node on one axis to a node on the other axis. Each regional node is represented by a single vertical and horizontal line of points corresponding to its connecting edges to other nodes. The resorting of the adjacency matrix based on the nodal magnitude order of the Fiedler vector provides insight as to the modularity of the underlying connective associations. Thus, three partially separate modules of edges are detected in the Fiedler resorted adjacency matrix of the PD33 whole brain signal (*bottom right*).

**
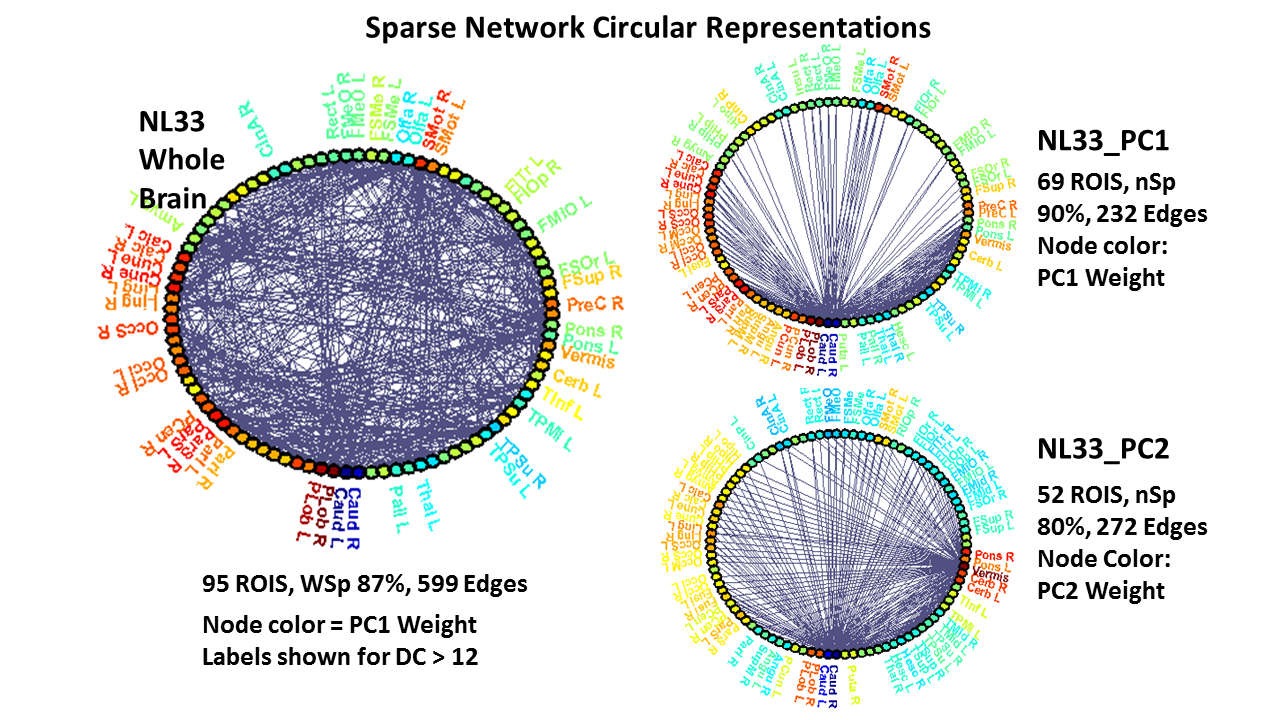

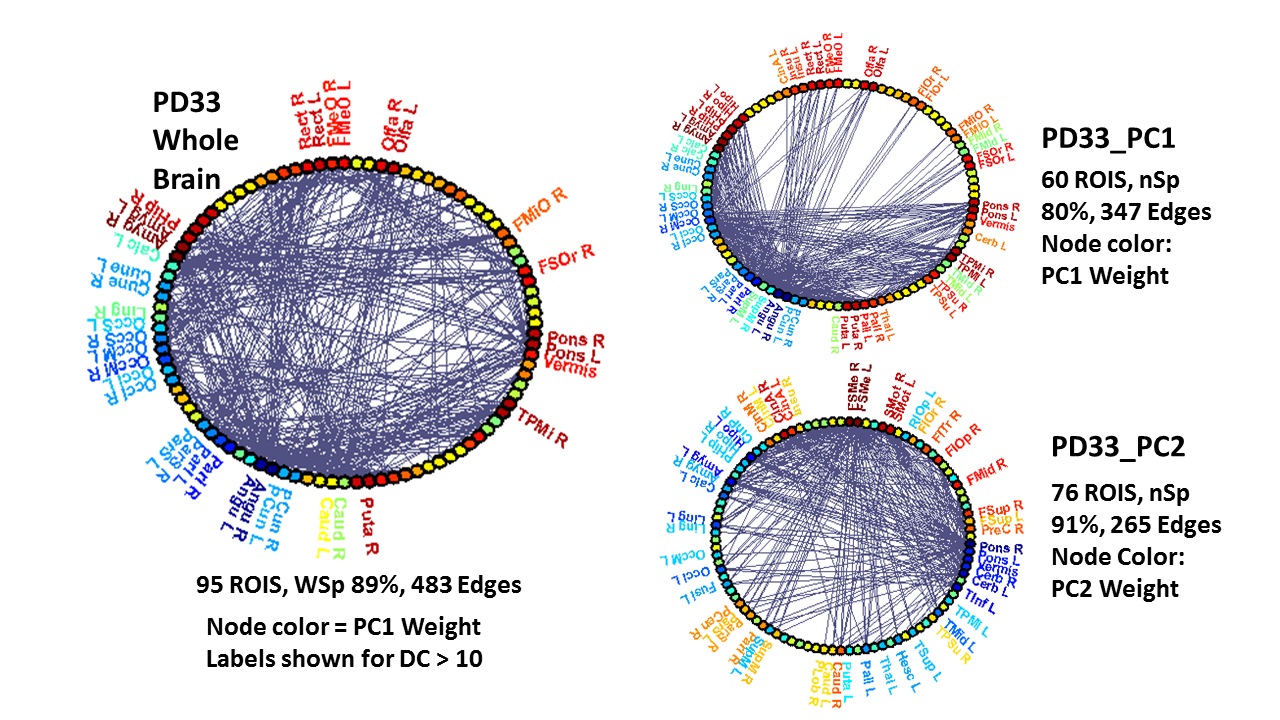
**

**Fig. S4. Sparse network circular representations of original sample data.** *Left:* Whole brain GLASSO derived nodal connections for fully connected networks at maximum sparsity are displayed in circular nodal configurations for the NL33 healthy group (*top left*) and the PD33 disease group (*bottom left*) original data set. The nodal colors correspond to the regional weights of the PC that exhibits the highest absolute value correlation to EC vector weights. Labels are shown for nodes with greater than the mean number of connections (degree weight DC > mean) *Right:* Sparse network connections are depicted for the partition layers of the first two PCs for the NL33 group (*top right*) and PD33 (*bottom right*) derived at specific values of the GLASSO penalty parameter. All connected nodes are labeled. The number of ROI nodes, edges and corresponding sparsity is indicated on each graph. The whole brain configurations appear to incorporate elements of each of its component PCs.


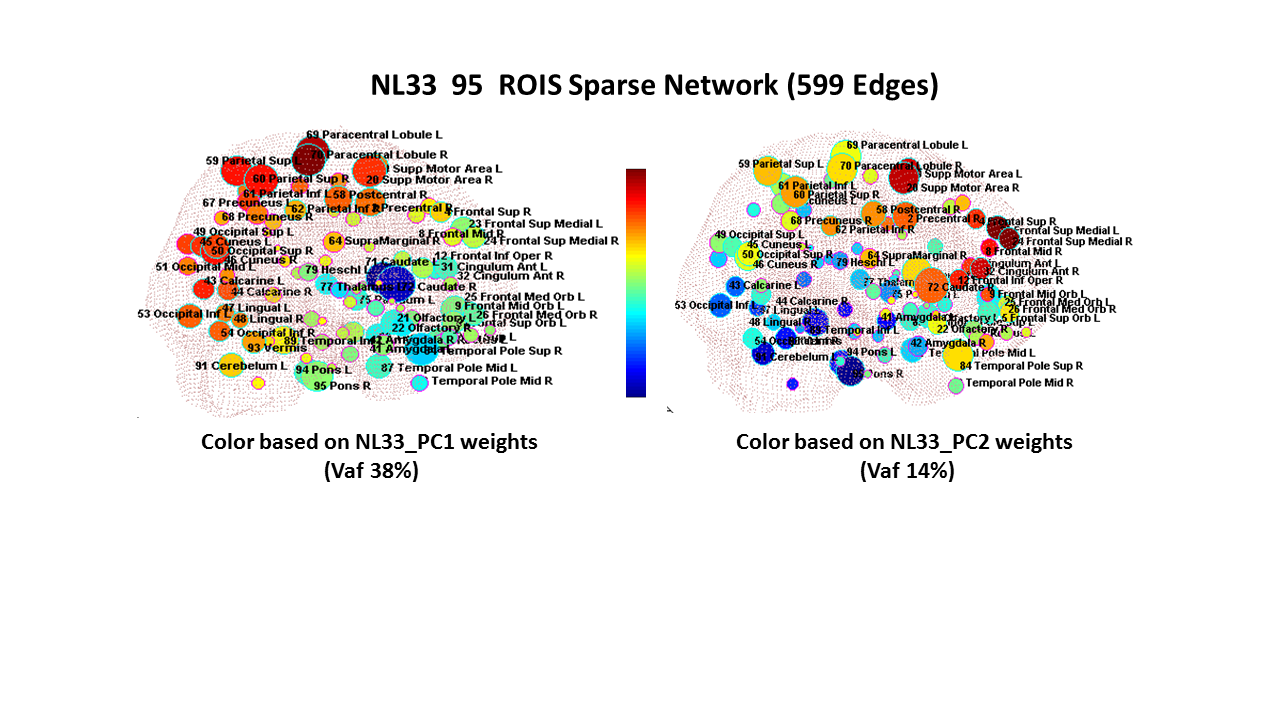


**Fig. S5.** **PC1 and PC2 color representations of the NL33 whole brain sparse network.** The NL33 original sample whole brain sparse network obtained at maximum sparsity that maintained full regional connectivity (95 ROIs, 599 edges) is displayed in regional colors that correspond to the weight of each node for the first two overlapping dominant PCs (NL33_PC1, 38% vaf; NL33_PC2, 14% vaf). Nodal diameter is set to correspond to the EC of the sparse network that is identical in both displays. *Left:* Correlation of EC with NL33_PC1 absolute and signed region weights was high (rEC=0.72, rECsigned=0.90, p<0.001). *Right:* For NL33_PC2 correlation was lower but also significant (rEC=0.36, rECsigned=0.83, p<0.001).


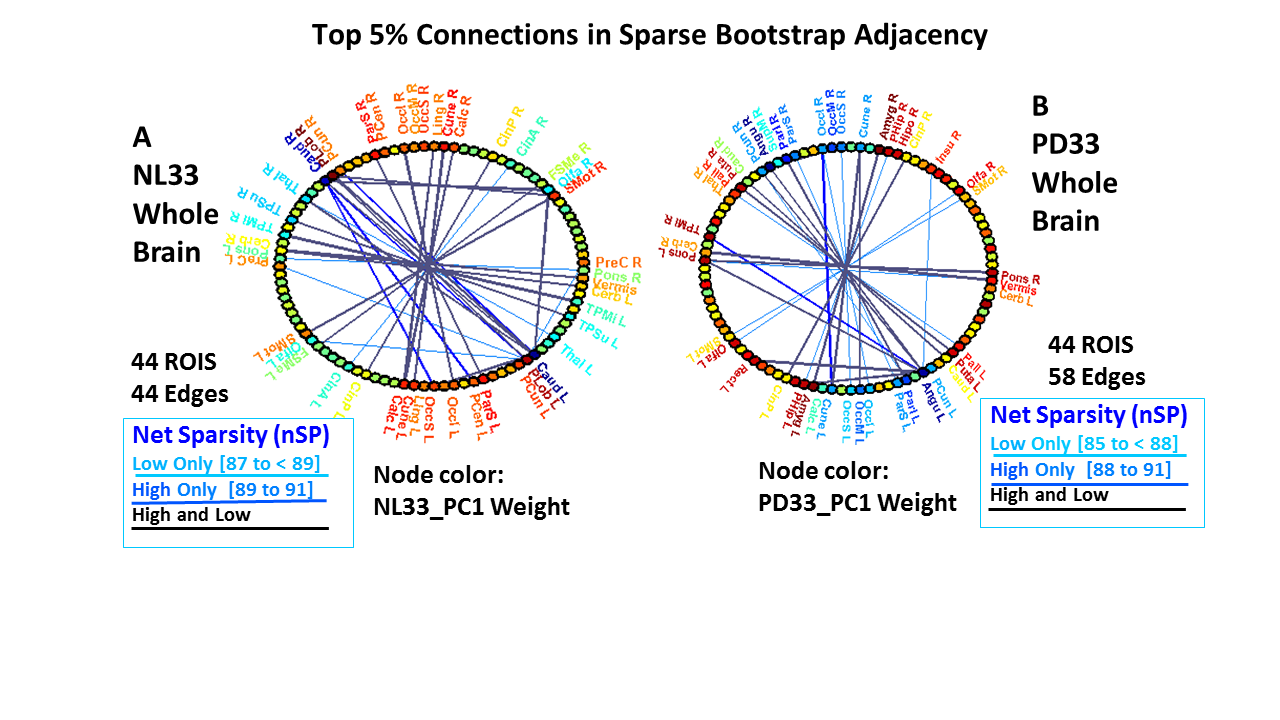

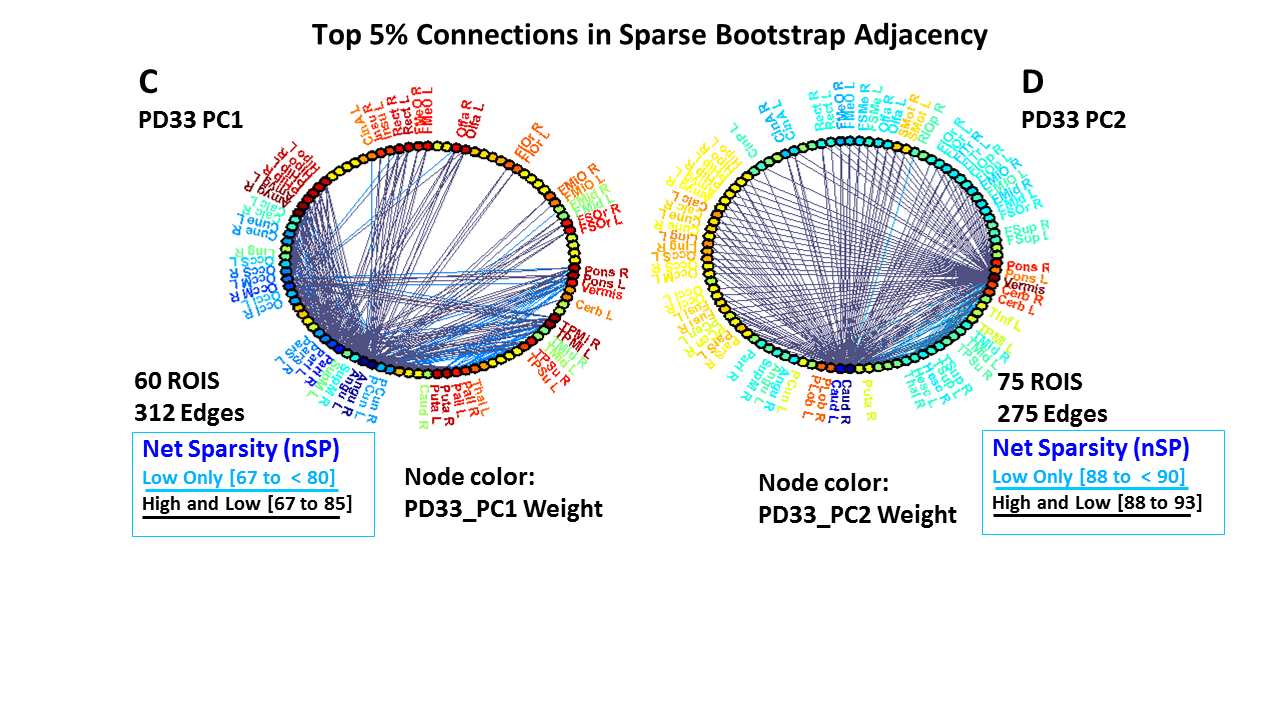


**Fig. S6.** Top 5% of sparse bootstrap adjacency. Circular displays corresponding to 3D displays of the main text Fig 6. See also Fig. 6 legend.

**A:** NL33 Whole Signal 200 samples, Total 44 edges, node color: NL33_PC1

Lower range wSP: 87% to <95%, nSP: 87% to <89%, 618 cases, Top 5%: (37 edges, light blue and black for 29 edges also included in the higher sparsity range)

High range wSP: 89% to 95%, nSP: 89% to 91%, 838 cases, Top 5%: (36 edges, blue and black for 29 edges also included in the lower sparsity range)

**B:** PD33, Whole Signal 200 samples, Total 58 edges, node color: PD33_PC1

Lower range wSP: 85% to 95%, nSP: 85% to <88%, 723 cases, Top 5%: (56 edges, light blue and black for 38 edges also included in the higher sparsity range)

High range wSP: 88% to 95%, nSP: 88% to 91%, 1396 cases, Top 5%: (40 edges, dark blue and black for edges also included in the lower sparsity range)

**C:** PD33_PC1 Partition, 500 samples, Total 312 Edges, node color: PD33_PC1

Lower range wSP: 85% to < 92%, nSP: 67% to < 80%,535 cases, Top 5% (312 edges, light blue and black for 224 edges also included in the higher sparsity range)

High range wSP: 92% to 95%, nSP: 80% to 85%, 958 cases, Top 5%: (224 edges, black,)

**D:** PD33_PC2 Partition, 400 samples, Total 275 Edges, node color: PD33_PC2

Lower range wSP: 90% to <94%, nSP: 88% to 90%, 788 cases, Top 5% (275 edges, light blue and black for edges also included in the higher sparsity range)

High range wSP: 94% to 96%, nSP: 90% to 93%, 203 cases, Top 5% (186 edges, black)

**
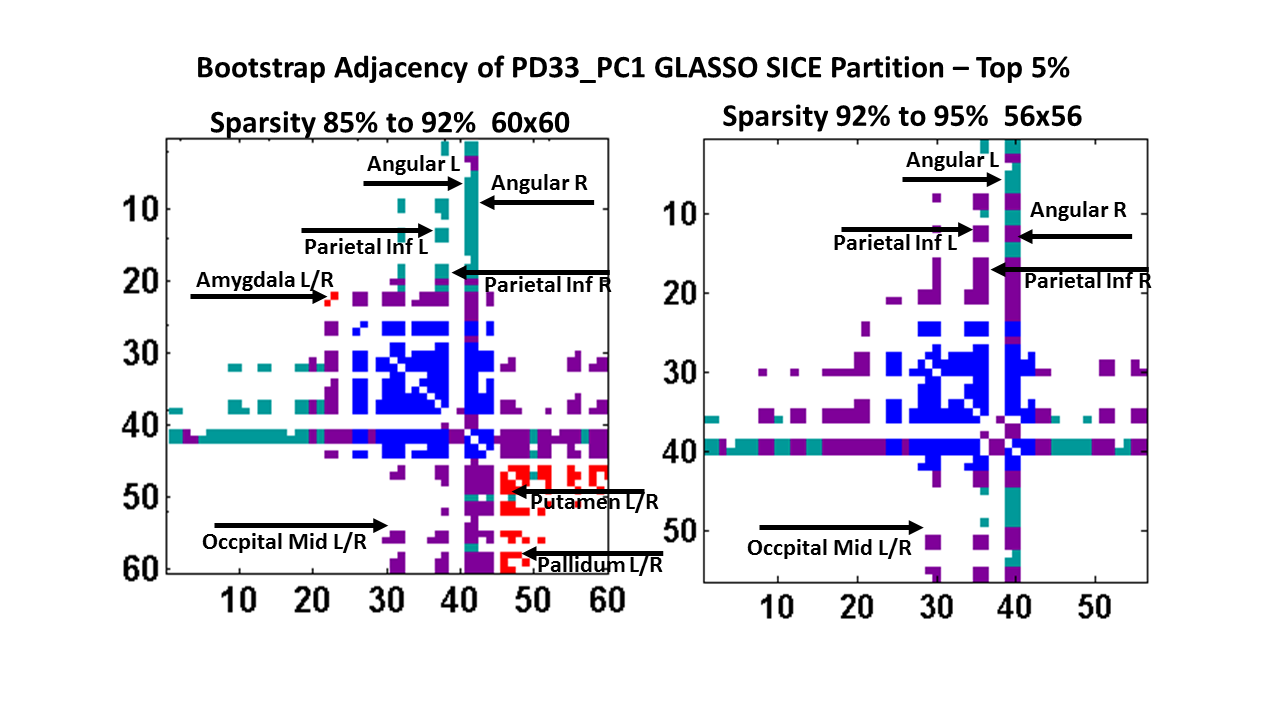
**

**Fig. S7. PD33_PC1 partition adjacency matrix for** **top bootstrap connections.** Composite PD33_PC1 partition adjacency matrix for the fully connected networks of edges present in 95% or more of the adjacency matrices within the 85% to less than 92% (*left*) wSP ( 67% to 80% nSP) and 92% to 95% (*right*) wSP (80% to 85% nSP) range of 500 bootstrap samples. Axes are in atlas index order*. Left:* Top 5% of edges present in 95% or more cases for 60 interconnected nodes*. Right:* Top 5% of edges present in at least 95% of cases for 56 interconnected nodes. The point elements of these matrices (edges) are colored based on whether they connect high EC (EC≥1std) nodes and whether the polarity of the two nodes within the associated PD33_PC1 vector was positive or negative. Thus, connections linking high EC negative nodes are shown in blue; connections linking positive to negative high EC nodes are shown in magenta. Connections between low EC nodes normally depicted in yellow are not apparent in these displays and connections between high EC positive nodes, shown as red are only apparent in the lower sparsity range (*left*) display for several regions including Putamen L/R, Pallidum L/R and Amygdala L/R connections. Vertical and horizontal lines of points consist of the edges (connecting points) of single prominent nodes that act as local (Amygdala, Putamen) or broader range hubs (Angular, Inf Parietal, Mid Occipital) connecting multiple other nodes.

**
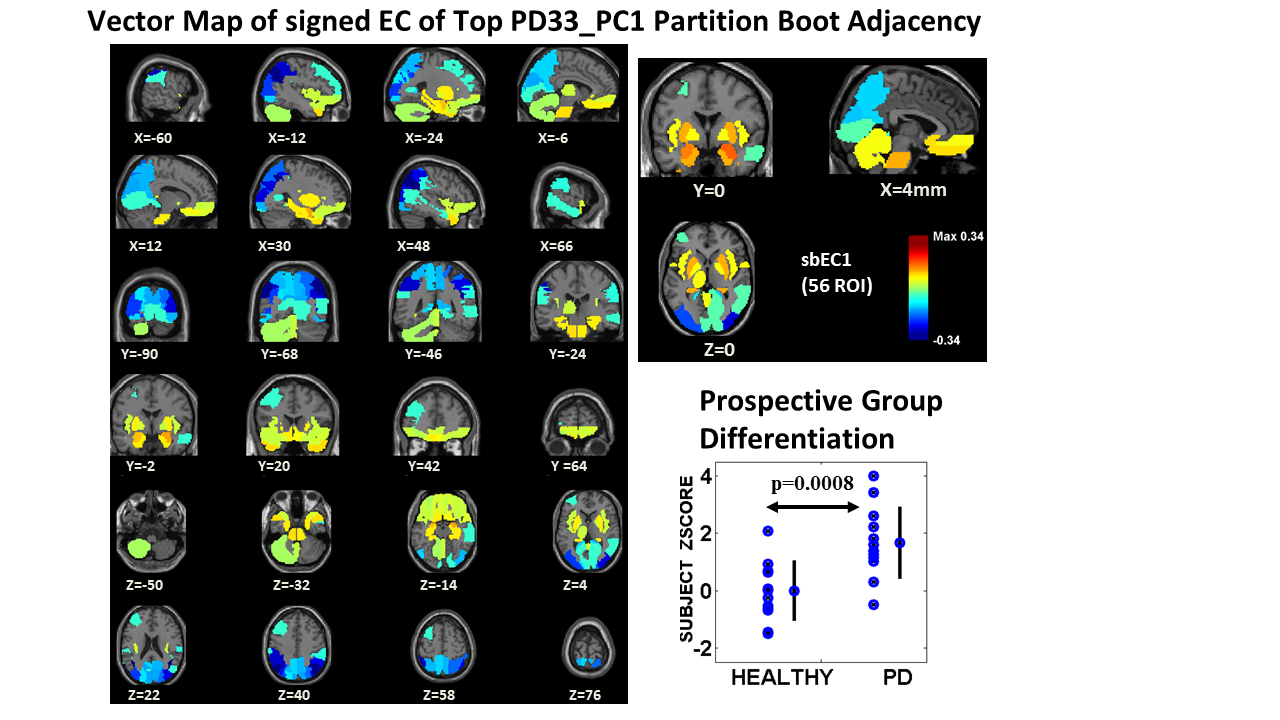
**

**Fig. S8. Signed EC of the adjacency matrix of the PD33_PC1 partition significant bootstrap connections.** *Left and top right:* Vector map representation of the signed EC (sbEC1) of the composite adjacency matrix of the top 5% of connections found in bootstrap assessment of connected subnetworks of the PD33_PC1 partition data. Five hundred bootstrap samples were evaluated for a variable range of penalty values in GLASSO analysis for which the adjacency sparsity range was 89% to 95% wSP (67% to 85% nSP). The adjacency matrix consisted of 56 ROI nodes strongly connected by 224 edges that were present in 95% or more of 1252 adjacency matrices satisfying the sparsity criteria. The Pearson’s correlation of the sbEC1 vector weights with the PD33_PC1 vector weights was r=0.97, r^2^=0.9, p<0.001. *Bottom right:* Prospective group differentiation of sbEC1 pattern scores for the PD13 early PD group and the healthy NL13 group (p=0.0008, AUC=0.88).


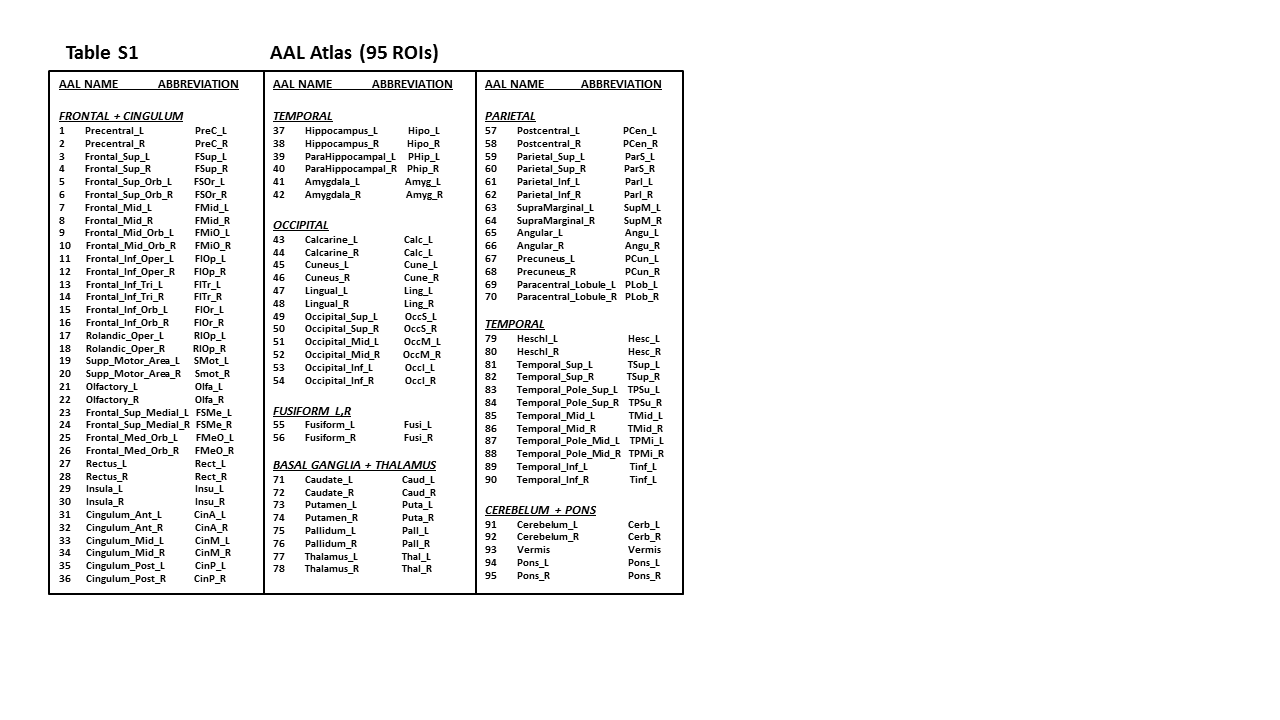
AAL Atlas numbers and names for 95 ROIS used in the analysis and abbreviations used in the text.


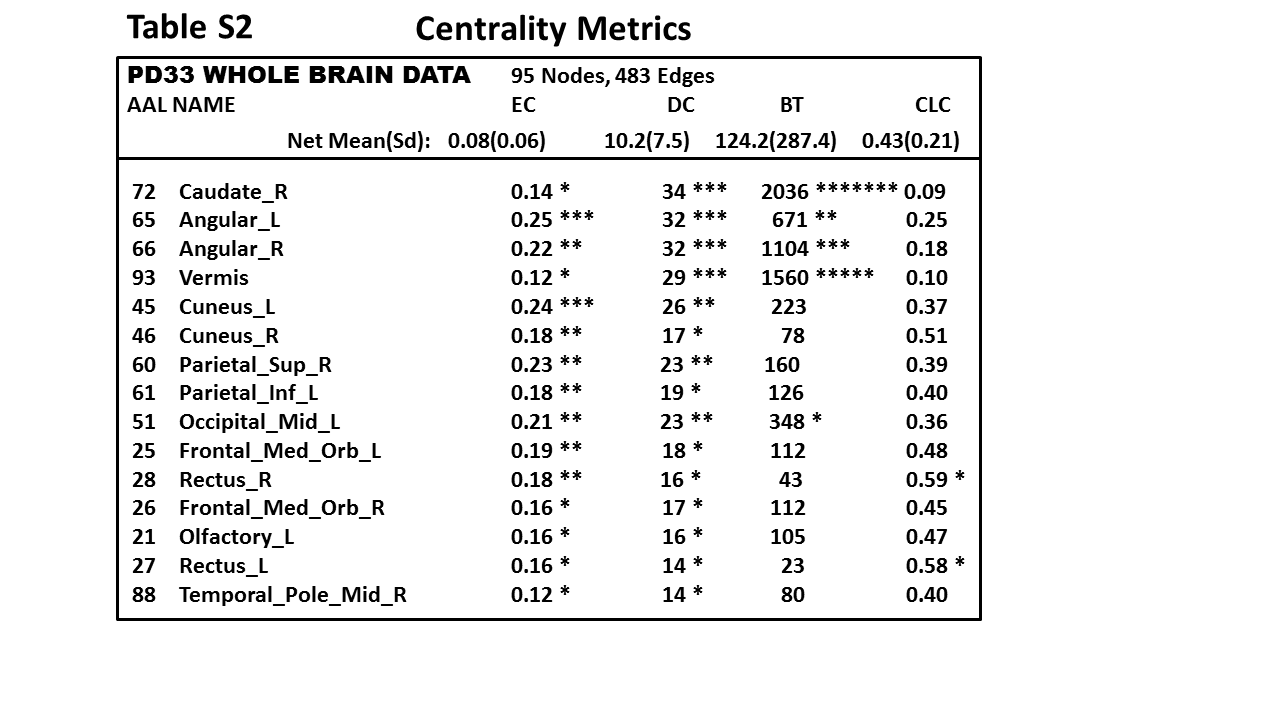

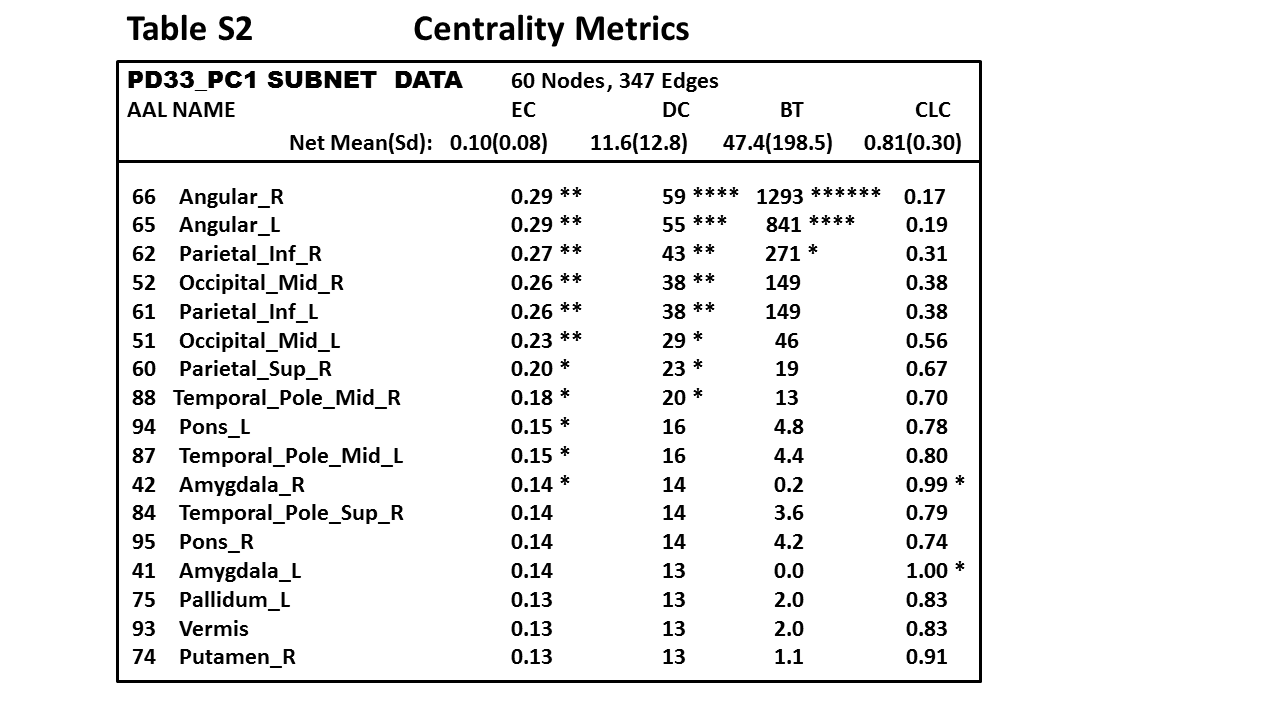

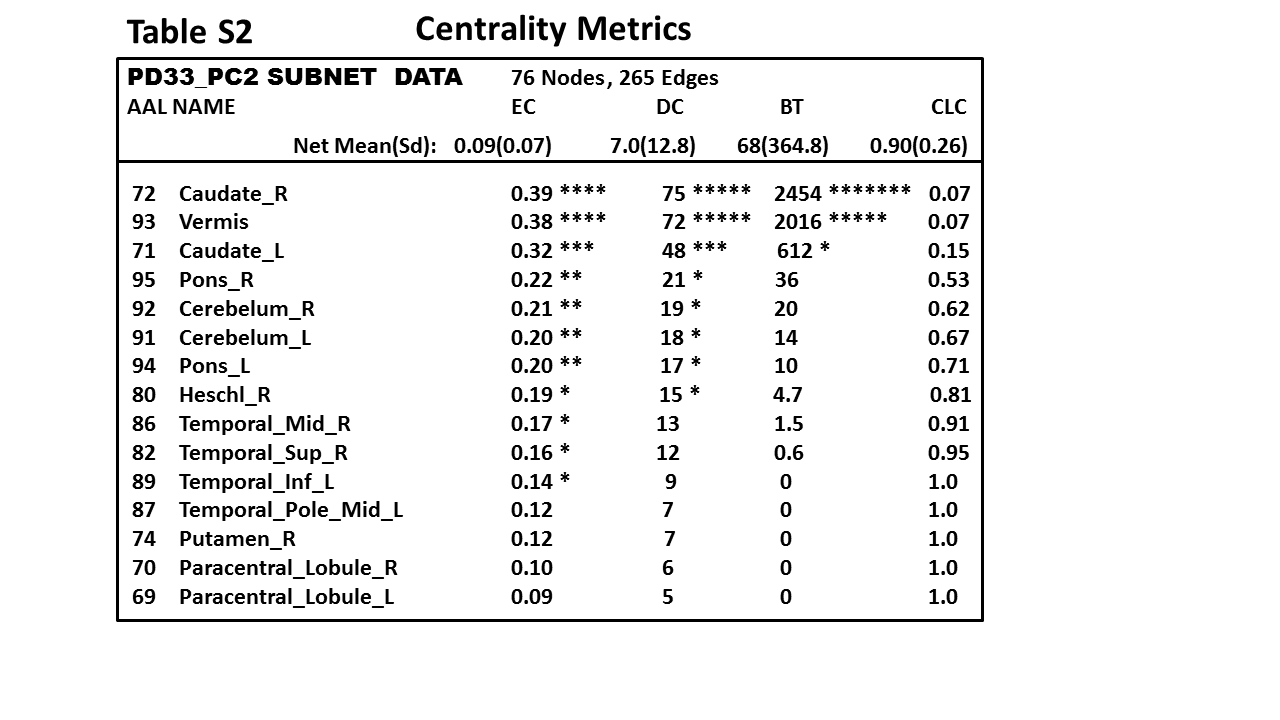

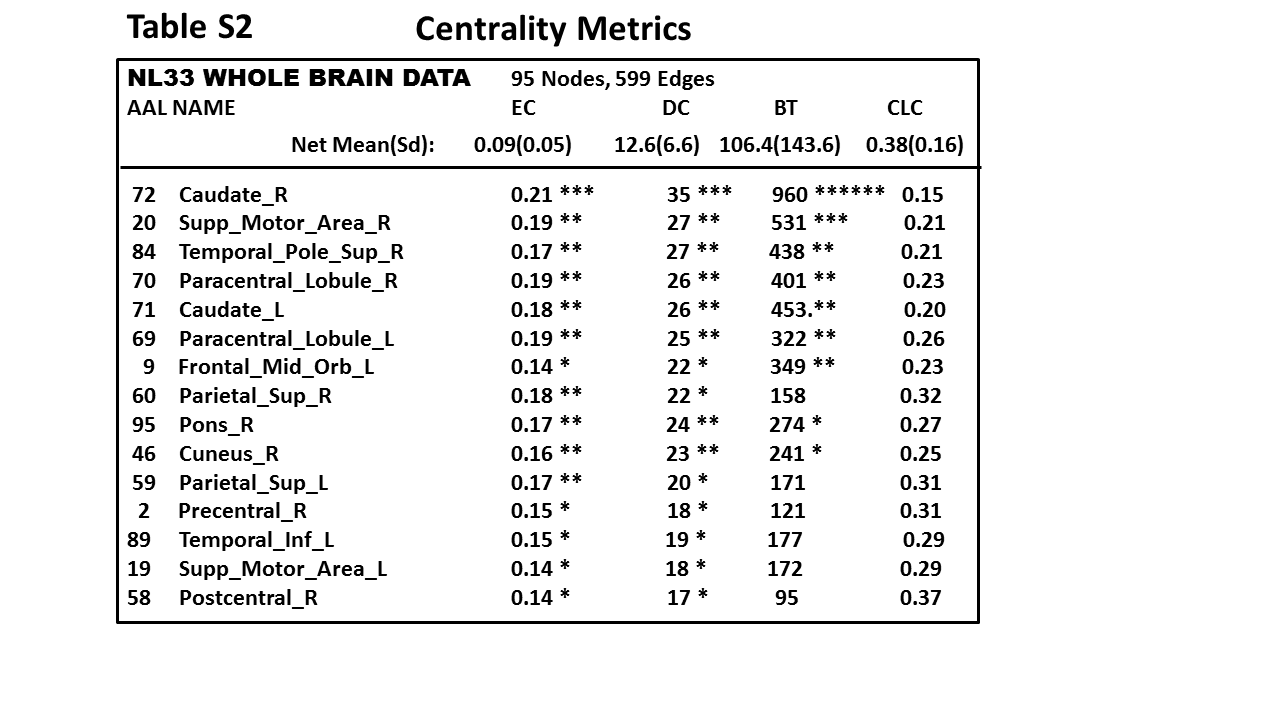

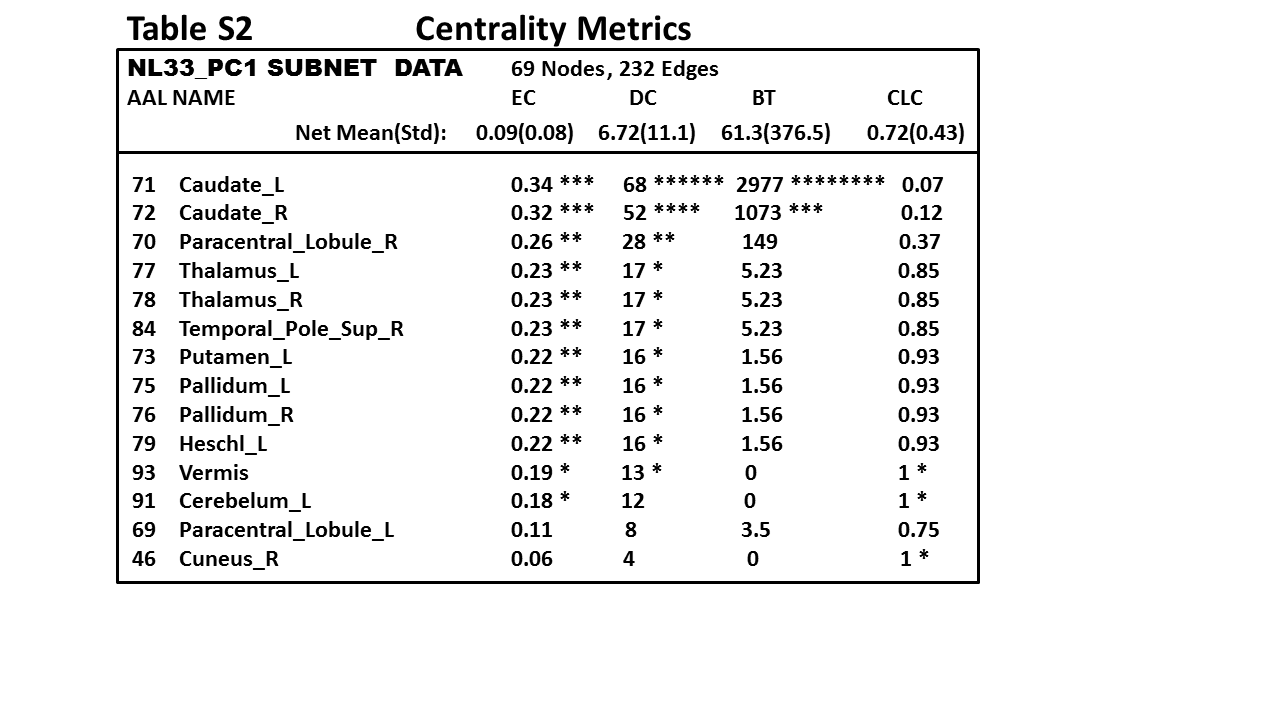

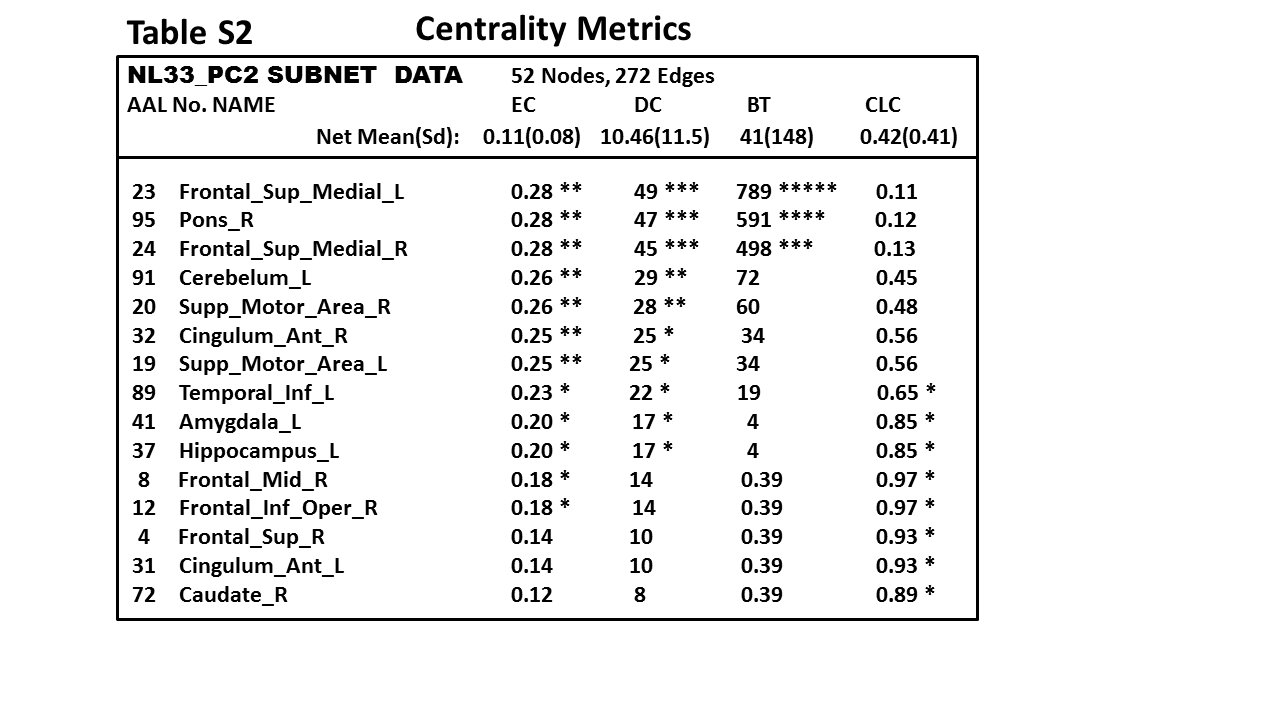


High degree (DC) and high eigenvector centrality (EC) nodes and their associated centrality metrics for DC, EC, betweenness (BT) and clustering coefficient (CLC) are listed for each of the whole brain and PC subnet configurations of the original data set. The number of nodes and edges in each network and the mean and standard deviation (std) of the values for each of the measures is noted in the heading. Nodes for which values are greater than one or more standard deviations above the mean are designated as hubs, with the number of standard deviations denoted by asterisks (*) adjacent to each value. Non-normalized values are reported for DC and BT.
